# Trends in mental health clinical research: Characterizing the ClinicalTrials.gov registry from 2007–2018

**DOI:** 10.1371/journal.pone.0233996

**Published:** 2020-06-05

**Authors:** Joshua R. Wortzel, Brandon E. Turner, Brannon T. Weeks, Christopher Fragassi, Virginia Ramos, Thanh Truong, Desiree Li, Omar Sahak, Hochang Benjamin Lee

**Affiliations:** 1 Department of Psychiatry, University of Rochester, Rochester, NY, United States of America; 2 Department of Internal Medicine, Kaiser Permanente Santa Clara, Santa Clara, CA, United States of America; 3 Department of Gynecology and Obstetrics, MGH, Harvard University, Boston, MA, United States of America; 4 Department of Psychiatry and Behavioral Sciences, Stanford University, Stanford, CA, United States of America; University of Rennes 1, FRANCE

## Abstract

While the epidemiologic burden of mental health disorders in the United States has been well described over the past decade, we know relatively little about trends in how these disorders are being studied through clinical research. We examined all US interventional mental health trials submitted to ClinicalTrials.gov between October 1, 2007 and April 30, 2018 to identify trends in trial characteristics, comparisons with non-mental health trials, and trial attributes associated with discontinuation and results reporting. International data were excluded to minimize potential confounding. Over this period, mental health and non-mental health trials grew at similar rates, though Industry and US government-funded trials declined and academic medical center/hospital/other (AMC/Hosp/Oth) funded trials grew faster in mental health research. The proportion of trials with safeguards against bias, including blinding and oversight by data monitoring committees (DMCs), decreased. This occurred during growth in the proportion of trials studying behavioral and non-pharmacological interventions, which often cannot be blinded and do not require DMC oversight. There was concurrent decline in pharmaceutical trials. There was significant growth in trials studying Non-DSM (*Diagnostic and Statistical Manual-5*) conditions (e.g. suicidality and wellness), as well as substance use, anxiety, and neurocognitive disorders. One in 12 trials was discontinued. Trial discontinuation was associated with industry and AMC/Hosp/Oth funders, pharmaceutical interventions, and lack of DMC oversight. Only 29.9% of completed trials reported results to the registry. Decreased results reporting was associated with behavioral interventions, phase 1 trials, and industry and AMC/Hosp/Oth funders. The main implications of these data are that funding is shifting away from traditional government and industry sources, there is increasing interest in non-pharmacological treatments and Non-DSM conditions, and there are changing norms in trial design characteristics regarding safeguards against bias. These trends can guide researchers and funding bodies when considering the trajectory of future mental health research.

## Introduction

As of 2001, the World Health Organization published that one in four people worldwide suffers from a mental health disorder across his or her lifetime, and in the United States, 46.6 million people currently suffer from a mental health disorder [1, 2]. As of 2016, mental health and substance use disorders accounted for 206.5 million disability-adjusted life years (DALYs), or 8.6% of all DALYs worldwide [3]. As of 2013, 16.7% Americans were prescribed a psychotropic medication, and in that year alone the United States spent $187.8 billion on treating mental health and substance use disorders, only trailing behind expenses to treat diabetes, cardiovascular disease, and back pain [4, 5]. However, while significant attention has been paid to the epidemiological impact of mental health disorders, relatively little focus has been placed on characterizing trends in how the research community has responded through clinical research. Specifically, there has been a gap in the literature studying changes in trial features, such as trial funders, trial design, targeted disorders, and interventions types.

To address the need for a public resource that could be used to identify and analyze clinical research, as well as the conditions, diseases, and interventions being researched and how they are studied, the National Institutes of Health created the ClinicalTrials.gov registry in 2000 [6]. In 2007, in accordance with Section 801 of the Food and Drug Administration Amendments Act (FDAAA), all United States non-phase 1 trials involving US Food and Drug Administration (FDA) regulated drug and biological products, as well as non-feasibility trials of FDA regulated devices, were mandated to report to a clinical trials registry [7]. Since then, ClinicalTrials.gov has grown to become one of the largest international registries for clinical research, and currently it contains detailed information on more than 335,000 clinical studies conducted in over 200 countries. It has been analyzed in over 300 research articles to characterize the landscape of clinical research [7, 8], and these articles have shed light on publication bias [9], noncompliance with trials registration [10], and selective reporting [11]. They have also identified trends across medical research [12] and within individual fields [12–14] of how trials are funded and designed, as well as what medical disorders and interventions are studied.

The field of mental health has only just begun to utilize ClinicalTrials.gov to help answer such questions regarding its clinical trials. The earliest large scale effort was a review of trials between 2007–2010 by Califf and colleagues, which examined Mental Health trials along with trials in Cardiology and Oncology [12]. In 2017, Anand and colleagues identified all trials in the registry relevant to bipolar disorder and observed a disproportional growth of trials in pediatric populations and in trials studying transcranial magnetic stimulation as treatment [15]. In 2019, Arnow and colleagues published a paper in which they identified all mental health trials in the registry from 2007 to 2014, and they evaluated differences in trial characteristics, such as trial design, disorders studied, and interventions tested as stratified by funder type [16]. Their findings included identifying that universities and hospitals funded the majority of mental health trials (64%), followed by governmental agencies (25.6%) and industry (21.5%). They found that the majority of trial characteristics tended to differ by funder. For example, more industry trials studied pharmacotherapy (95.2%) than behavioral interventions (0.9%), whereas government funders studied more behavioral interventions (60.6%) than pharmacotherapies (25.6%). They found that the most commonly studied conditions were mood disorders (40.0%), and that the majority of trials enrolled fewer than 100 participants, were randomized, and employed some form of blinding. Industry-funded trials were notable for enrolling more participants, as well as for using data monitoring committees and masking methods less often.

However, there are several questions that Arnow and colleagues left unanswered. First, while they provided a snapshot of mental health trials registered from 2007 to 2014, they did not provide temporal trends of how these trials changed to assess where the field may be heading. Their assessment of trials also ends in 2014, which limits extrapolation to more contemporary trends. In 2014, the Research Domain Criteria (RDoC) initiative instituted by the National Institute of Mental Health (HIMH) was just starting to shape the landscape of what was studied by government-funded trials [17]. Multiple large pharmaceutical companies had also just dramatically downsized their research arms for mental health, and it remains unclear how these changes shaped the clinical trials landscape in the interim [18, 19]. Second, they did not assess how mental health trials differed from non-mental health trials in the registry, which can provide context for interpreting results. Third, while they provided some information about the mental health disorders studied in these trials, the disorder categories they used were broad and often grouped multiple *Diagnostic and Statistical Manual-5 (DSM-5*) classifications into one category (e.g. “Mood” as provided by ClinicalTrials.gov includes both depressive disorders and bipolar and related disorders). This limits a more nuanced interpretation of how the study of mental health disorders has changed. Lastly, while other fields have utilized the ClinicalTrials.gov database to assess results reporting and trial discontinuation, this has not been done within mental health research.

This study sought to provide clarity for these lingering questions. We performed an analysis of the entire portfolio of United States mental health clinical trials registered in ClinicalTrials.gov from 2007 to 2018. We examined time trends in funder type, study design, disorder categories, interventions, and other trial features by calculating annual growth rates and by stratifying trials into two time periods (early [2007–2012] and late [2013–2018]). We manually parsed the disorders studied using *DSM-5* classifications to gain a more nuanced understanding of how the field has changed. We compared mental health and non-mental health trials to gain further perspective on these changes. We also conducted logistic regression and survival analysis to investigate the characteristics of mental health trials associated with trial discontinuation and results reporting to the ClinicalTrials.gov registry.

## Methods

### Data selection and classification

We downloaded records on April 30, 2018 for all 274,029 trials submitted (i.e. some trials may have been submitted but not yet posted) as of April 30, 2018 to ClinicalTrials.gov using the Aggregate Analysis of ClinicalTrials.gov (AACT), a relational database of publicly available ClinicalTrials.gov data [20]. We selected for interventional trials (a category provided within ClinicalTrials.gov) submitted to the registry on or after October 1, 2007 to coincide with the passing of the Food and Drugs Administration Amendments Act (FDAAA) on September 27, 2007 [7]. ClinicalTrials.gov defines ‘interventional’ trials as “studies in human beings in which individuals are assigned by an investigator based on a protocol to receive specific interventions” [12]. We identified trials relevant to mental health using the Medical Subject Heading (MeSH) terms and Disease Condition terms provided for each trial, as previously described [12–14, 16]. A psychiatrist reviewed the list of all MeSH and Disease Condition terms in the ClinicalTrials.gov registry, and those terms deemed relevant to mental health were selected and reviewed by another physician (S1 Table). All trials selected through this process were divided among six psychiatrists who manually reviewed the official title and study description to: (i) exclude trials not relevant to mental health; and (ii) categorize the remaining trials according to the disorder index categories in the Section II Diagnostic Criteria and Codes provided by the *DSM-5* (S2 Table) [21]. Trials that identified disorders by *DSM-IV* diagnostic nomenclature were reclassified using equivalent terms in the *DSM-5*. All psychiatrists reviewed a sample of the same 250 trials to ensure agreement on the labeling criteria. Trial categorizations with any ambiguity were marked and reviewed by another psychiatrist. When appropriate, trials were assigned to more than one *DSM-5* category. Trials that did not clearly match any *DSM-5* category (e.g. stress, burnout, or suicide) were marked “Non-DSM” conditions. Because requirements for registration and reporting of results to trial registries vary by country, only trials with research sites exclusively within the United States were included in this analysis.

### Changes to the initial protocol

We developed a protocol for our analysis that was submitted with our manuscript. It was not pre-registered. We subsequently made several changes to this protocol (detailed in S3 Table). These changes resulted from the helpful direction of our reviewers and in response to the work by Arnow and colleagues, whose paper was published after completion of our initial analysis [16]. In brief, there were eight changes to our initial protocol:

1. We initially analyzed both US and international studies, as this had been done in some other analyses of the registry [7, 10, 14]. However, because trial registration practices differ significantly by country, there was concern that inclusion of international trials potentially confounded our results (i.e. it was difficult to determine whether observed trends were due to true differences in trial characteristics by region or differences in trial registration). Therefore, our revised analysis was limited to only US trials.
2. Our initial analysis excluded the ClinicalTrials.gov funder category US Fed, which has been done in some other analyses since US Fed comprises only 3.5% of trials in the registry [14]. However, we subsequently combined US Fed-funded trials with NIH-funded trials to form a new funder category called ‘US Govt’ to better capture changes in US government-funded trials.
3. Our initial analysis included a table of trial characteristics stratified by funder type. However, Arnow and colleagues performed a very similar analysis for mental health trials in ClinicalTrials.gov from 2007–2014 with similar results. Rather than duplicate their work, we discussed other findings from our analysis concerning comparisons of mental health and non-mental health trials in the ClinicalTrials.gov registry.
4. Our initial analysis included enrollment as a trial characteristics to assess over time; however, many trials in the registry only reported anticipated/estimated enrollment. Our reviewers advised that estimated enrollment is an unreliable metric, as many trials do not meet this projected enrollment number. Because studying only the trials that reported actual enrollment would introduce significant bias into our analysis, we removed almost all discussion of enrollment from our revised analysis.
5. We initially clustered Phase 1/2 and Phase 2/3 trials under the phase category ‘Not Applicable;’ however, our revised analysis grouped these trials with Phase 2 and Phase 3 trials, respectively, as these trials were deemed to have ultimately reached Phase 2 and Phase 3 status.
6. Our initial analysis assessed results reporting within 2 years of trial completion to account for the 12-month reporting period and the mid-point of the available extension time provided for certain trials by the FDAAA Section 801 and the Final Rule [22]. However, our revised analysis included results reporting within the maximum extension time period provided for certain trials by the FDAAA 801 and the Final Rule (i.e. 3 years), as this was deemed to be a more robust analysis.
7. Our initial analysis did not include an assessment of interventions studies, though this was included in the revised analysis.
8. We included the citation of Arnow and colleagues in our revised protocol and manuscript, as their work is fundamental to contextualizing our study and several changes made to our revised analysis [16].

### Trial characteristics

We analyzed each trial along the following 13 dimensions:

1. Year of submission (dates ranged from 2007 to 2018). We divided our 127-month study period at the approximate midpoint into a 63-month early period (October 1, 2007 to December 31, 2012) and a 64-month late period (January 1, 2013 to April 30, 2018). Throughout the analysis, time of submission was assessed as a dichotomous variable using these groupings.
2. Primary objective of the intervention (categories included Treatment, Basic Science, Prevention, and Other). ‘Other’ was generated by combining the category Other in ClinicalTrials.gov with the categories Device Feasibility, Diagnostic, Health Services Research, Screening, and Supportive Care, which together made up 14.2% of trials.
3. Trial phase (categories included Phase 1, Phase 1/2–2, Phase 2/3–3, Phase 4, and Not Applicable). ‘Phase 1’ was generated by grouping the ClinicalTrials.gov categories Early Phase 1 and Phase 1. ‘Phase 1/2–2’ was generated by grouping the ClinicalTrials.gov categories Phase 1/2 and Phase 2. ‘Phase 2/3–3’ was generated by grouping the ClinicalTrials.gov categories Phase 2/3 and Phase 3. ‘Phase 4’ and ‘Not Applicable’ were taken directly from these corresponding categories in ClinicalTrials.gov. According to the definition provided by the National Library of Medicine, the label ‘Not Applicable’ is used to describe trials without FDA-defined phases, including trials of devices or behavioral interventions [23].
4. Number of arms (grouped by range: One, Two, or ≥Three). Number of arms was treated as a nominal variable using these groupings.
5. Blinding (categories included None, Single, and Double). The category ‘Blinding’ was generated from the category Masking in ClinicalTrials.gov.
6. Use of randomization (categories included No or Yes). This was taken directly from the categorization in ClinicalTrials.gov.
7. Oversight by a data monitoring committee (DMC) (categories included No or Yes). This was taken directly from the categorization in ClinicalTrials.gov.
8. Number of sites (categories included One, Two, Three–Ten, and >Ten). Of note, in the logistic and cox regressions performed as part of this study, these categories were further consolidated to ‘One’ or ‘≥Two,’ as trials with multiple sites were thought to share more in common than single site studies. Altogether, multi-site trials comprised 22.7% of trials. Number of sites was treated as a nominal variable using these groupings.
9. Funder (categories included Industry, Academic Medical Centers/Hospitals/Other [AMC/Hosp/Oth], and United States Government [US Govt]). The category ‘US Govt’ was generated from the ClinicalTrials.gov categories NIH and US Fed, as previously described [16]. Any trial with a sponsor or collaborator that was industry was classified as having an ‘industry’ funder, and any trial with ‘NIH’ or ‘US Fed’ sponsor or collaborating funders was classified as having a ‘US government’ funder. If trials sponsor/collaborators included both industry and US government funders (n = 112, 1.8% of trials), funding was labeled ‘industry,’ as we wanted to prioritize the involvement of industry in our analysis of trial characteristics. In their study, Arnow and colleagues identified that the majority of trials with funder labeled ‘Other’ were funded by universities or hospitals, with the remaining minority including consortiums, foundations, individuals, and community-based associations [16]. For this reason they renamed the ClinicalTrials.gov ‘Other’ funder category as ‘University or Hospital.’ We examined a random sample of 2,500 Other-funded trials in the database and identified 94.7% of these funders as academic institutions or hospitals. We believed the label Other ignores the dominant identity of these agencies and suggests greater heterogeneity than this category comprises. Therefore, we similarly renamed the ‘Other’ funders in our study as ‘Academic Medical Centers/Hospitals/Other.’ Given that Arnow and colleagues delineated trial characteristics by funder (of note, in their study they use ‘sponsor’ and ‘funder’ interchangeably), we assessed funder only as it related to time trends, results reporting, trial discontinuation, and intervention and disorder categories. When discussing the top fifty sponsors of mental health trials, we used the term ‘sponsor’ as defined by ClinicalTrials.gov: “Sponsor [is] the organization or person who initiates the study and who has authority and control over the study” [23]. For this reason ‘sponsor’ is used for Table 5 but elsewhere in the paper ‘funder’ is used.
10. Study status (categories included Complete, Ongoing, Stopped Early, or Unknown). The category ‘Stopped Early’ was grouped from the ClinicalTrial.gov study status categories Terminated, Withdrawn, or Suspended. The other categories match those in ClinicalTrials.gov.
11. Intervention (categories include Behavioral, Pharmaceutical, and Other). ClinicalTrials.gov provides data on multiple interventions including Behavioral, Drug, Device, Procedure, Dietary Supplement, Radiation, Biological, Genetic, and Other. Because several of these categories were used by only a small number of trials, and because we thought it was important to assess the number of trials studying more than one intervention type, we chose to consolidate these categories into three broader categories. Specifically, the category ‘Pharmaceutical’ was created to include the registry categories Drug, Dietary Supplement, and Biological. The category ‘Other’ was expanded to include the registry categories Device, Procedure, Radiation, Genetic, and Other. The category ‘Behavioral’ is the same as that reported in ClinicalTrials.gov.
12. Disorder categories (categories include Substance, Depression, Neurodevelopment, Trauma, Schizophrenia, Anxiety, Sleep, Bipolar, OCD, Feeding, Neurocognitive, Disruptive, Sexual, Personality, Somatic, Movement, Dissociative, Gender, Paraphilic, and Non-DSM). See above for how these trials were manually sorted into these categories and S2 Table for the disorders included in these categories. Trials were labeled with as many categories as were relevant, and consequently the percent of trials by disorder category sums to greater than 100%. In the logistic and cox regressions, each disorder category was evaluated as a binary covariate for the presence or absence of that category. Our models did not include covariates for the following groups due to their sparse usage (together comprising only 1.9% of trials): disruptive, sexual, personality, somatic, movement, dissociative, gender, and paraphilic disorders.
13. Results reporting (categories included Yes and No). See section below on results reporting.

### Time trend analysis

To facilitate our exploratory analysis of changes in trial characteristics over time, we separated trials into two time periods, as described above, and assessed for significant differences between periods. To summarize year-to-year changes in trial counts, we calculated average annual growth rates (AAGR) and compound annual growth rates (CAGR). The AAGR is calculated by taking the arithmetic mean of the percent change in a variable each year. While an accurate representation of the yearly growth of a variable, arithmetic means can be easily skewed by outlier values (i.e. dramatic fluctuations in growth). The CAGR is calculated by taking the final variable size compared to the initial variable size and approximating the average theoretical annual growth that would have been needed to reach the final size. Consequently, CAGR lessens the impact of large fluctuations in approximated growth rate. By comparing AAGR and CAGR, one can understand more thoroughly how a variable changed over time.

### Early discontinuation and results reporting

We assessed the early discontinuation of mental health trials in our sample. See ‘Study status’ under Trial Characteristics for how early discontinuation was determined from ClinicalTrial.gov study status categories. A total of 5,818 trials (92.3% of total) were included in this analysis, excluding trials that were withdrawn before initiation or if discontinuation status was unknown.

We examined results reporting to understand dissemination of trial results into the database; however, only a subset of trials are required by the FDAAA to report results into the database. We examined results reporting by 36 months after the trial primary completion date to coincide with FDAAA provisions that relevant trials report results within 12 months, with opportunities for an additional 24-month extension [24, 25]. Therefore, we restricted our reporting analysis to mental health trials that reached primary completion by April 30, 2015 (n = 2,223) to ensure a full 36-month reporting window. Because many trials report results outside of this window, we also separately analyzed results reporting without restrictions on the date.

### Statistical analysis

We summarized trial data using descriptive statistics. Because ClinicalTrials.gov does not mandate that certain optional fields be completed, approximately 5% of trials had missing dimensions, and, therefore, the total number of trials varies slightly between dimensions. The total number of trials reporting each trial characteristic is labeled in Tables 2, 3 and 4. Because only a small percentage of trials had missing data, these trials were excluded from the logistic and cox regressions. We assessed the statistical significance of monotonic trends over time (i.e. annual growth rates and compound annual growth rates) using post-hoc Mann-Kendall tests to test the null hypothesis that the number of trials did not change over time. We assessed independence between groups over ordinal time using the Cochran-Armitage test. All year-to-year analyses included only years with a full 12-month collection of data (2008–2017). We assessed for differences between the distributions of categorical variables using a two-sided Pearson χ2 test. All analyses were two-sided.

We performed time-to-event analysis of early trial stoppage with early discontinuation as events. We censored trials that reached completion without early stoppage or that remained ongoing at the cutoff for analysis (April 30, 2018). We visualized the relationship between trial duration and early discontinuation using Kaplan-Meier curves. We also performed Cox proportional hazards regression and provide individual and adjusted hazard ratios (aHR) for each trial characteristic (e.g. primary objective, trial phase, blinding, etc.). For trials that reached primary completion by April 30, 2015, we analyzed trial characteristics associated with results reporting to the registry using odds ratios (OR) and adjusted ORs (aOR) from univariate and multivariate logistic regressions, respectively.

We chose α = 0.001 as the level at which effect sizes represent meaningful trends and differences between groups given the large dataset and the risk of multiple hypothesis testing. We did not adjust for multiple comparisons in this exploratory analysis, and we expect approximately one out of every thousand tests to produce a significant result due to chance. All analyses were performed using the R statistical programming language, version 3.5.0 [26].

## Results

### Trial characteristics over time

**Fig 1:**
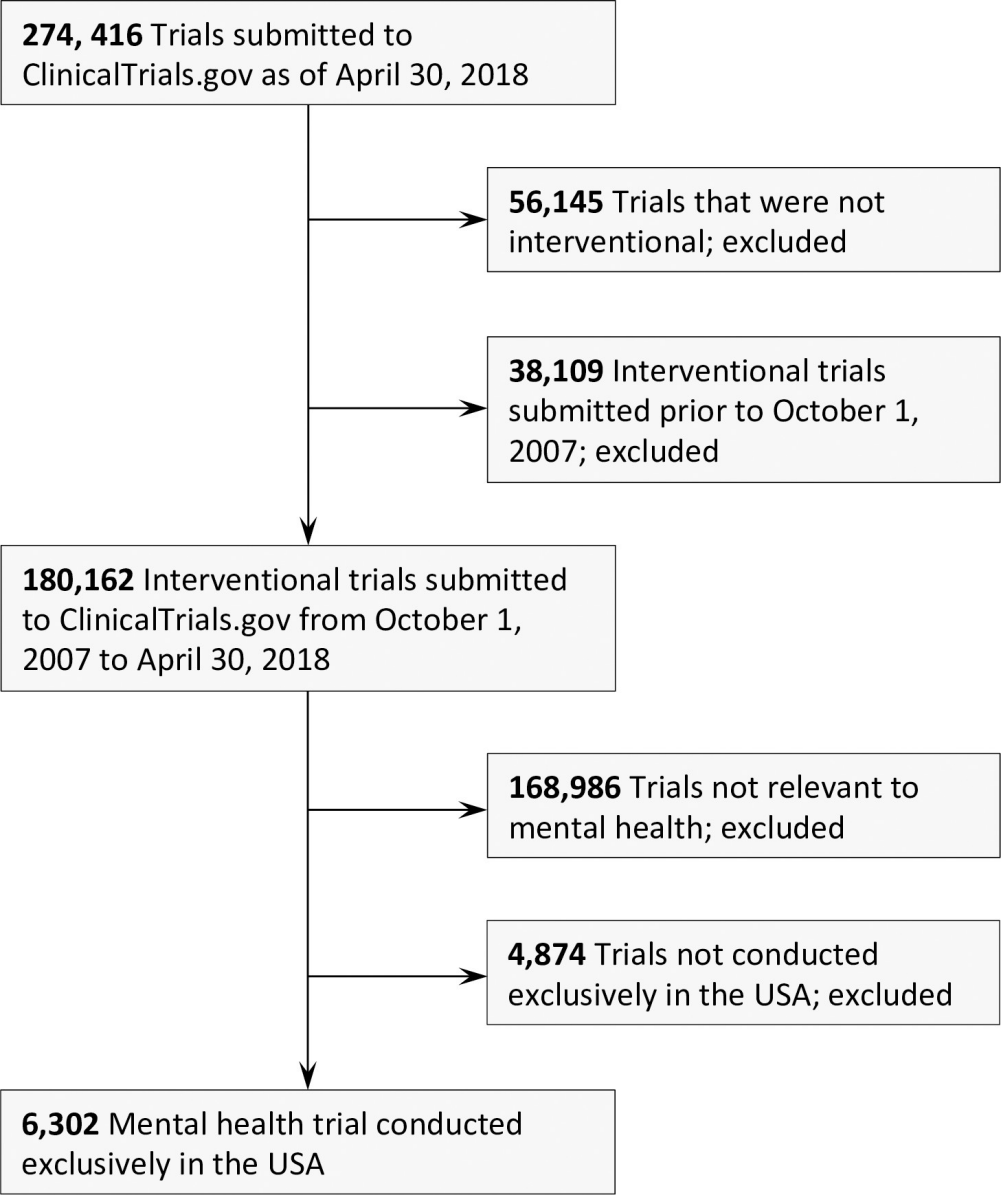
A flow diagram of inclusion of United States interventional mental health trials registered on ClinicalTrials.gov.

**Fig 2:**
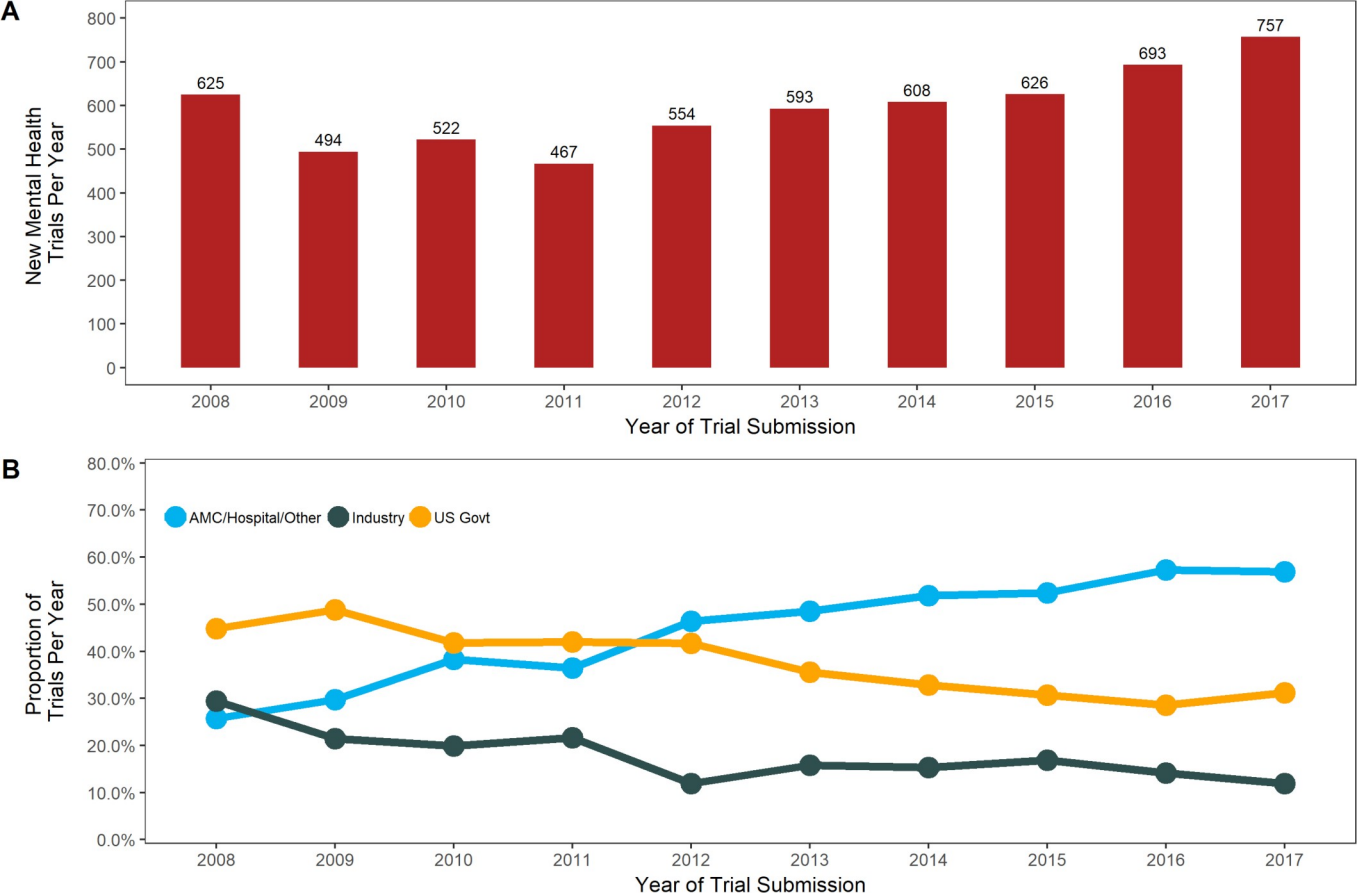
Characteristics of United States mental health clinical trials registered on ClinicalTrials.gov from January 1, 2008 to December 31, 2017. (A) The number of mental health clinical trials submitted to the registry by year. (B) Proportion of trials by year stratified by funders. ‘AMC/Hosp/Oth’ denotes Academic Medical Centers/Hospitals/Other. ‘US Govt’ denotes United States Government, which includes NIH (National Institutes of Health) and US Federal agency funders.

We identified 6,302 United States mental health trials, which comprised 56.4% (6,302/11,168) of all mental health trials and 10.2% (6,302/61,533) of all US interventional trials in the registry from October 1, 2007 to April 30, 2018 (Fig 1). From 2008 to 2017, the annual number of mental health trials increased from 625 to 757 (CAGR 2.2%, Mann-Kendall p = 0.012) (Fig 2A). This growth was roughly equivalent to that seen in non-mental health interventional trials in the United States during that period (CAGR 2.0%, Mann-Kendall p = 0.032) (Table 1). Industry-funded mental health trials (184 trials to 90; 29.4% of total to 11.9%; CAGR -9.6%, Mann-Kendall p = 0.012) and US government-funded mental health trials (280 trials to 236; 44.8% of total to 31.2%; CAGR -3.9%, Mann-Kendall p = 0.0013) decreased significantly. Trials funded by academic medical centers, hospitals, and other sources grew dramatically (161 trials to 431; 25.8% of total to 56.9%; CAGR 9.2%, Mann-Kendall p = 0.00035) and drove the overall growth of the field (Fig 2B). Similar trends occurred across all non-mental health interventional trials in the registry, though the decrease in industry and US government funders and the growth of academic medical center, hospital, and other funders were less pronounced (Ind CAGR -4.7%, Mann-Kendall p = 0.0013; US Govt -2.3%%, Mann-Kendall p = 0.0042; AMC/Hosp/Oth CAGR 6.4%, Mann-Kendall p = 0.00017).

**Table 1:** Average and compound annual growth rates of all United States mental health and non-mental health trials registered in ClinicalTrials.gov from January 1, 2008 to December 31, 2017.

	Mental Health Trials				Non-Mental Health Trials			
	AAGR	CAGR	M-K p-val	C-A p-val	AAGR	CAGR	M-K p-val	C-A p-val
**Total**	2.8%	2.2%	0.012	-	2.3%	2.0%	0.032	-
**Funder**								
Industry	-7.0%	-9.6%	0.012	<0.0001	-4.5%	-4.7%	0.0013	<0.0001
AMC/Hosp/Oth	9.8%	9.2%	0.00035		6.5%	6.4%	0.00017	
US Govt	-3.6%	-3.9%	0.0013		-2.2%	-2.3%	0.0042	

‘AMC/Hosp/Oth’ denotes Academic Medical Centers/Hospitals/Other. ‘US Govt’ denotes United States Government, which includes NIH (National Institutes of Health) and US Federal agency funders. AAGR denotes average annual growth rate. CAGR denotes compound annual growth rate. M-V p-val denotes Mann-Kendall p-value, and C-A p-val denotes Cochran-Armitage p-value.

There were multiple significant changes in trial characteristics between the early and late time periods (i.e. early [2007–2012] and late [2013–2018]) (Table 2). Interventional trial objectives shifted towards Prevention (7.4% to 10.4%), Basic Science (4.5% to 7.4%), and Other objectives (10.5% to 17.2%, including Health Services and Supportive Care), and away from Treatment (77.7% to 65.0%) (all: p<0.0001). There was a significant increase in the proportion of trials that did not have applicable phase designations (41.6% to 63.0%; p<0.0001), trials with single or no blinding (24.5% to 30.0% and 39.5% to 43.1%, respectively; both: p<0.0001), trials without oversight by data monitoring committees (DMC, 51.3% to 58.7%; p<0.0001), and trials conducted at only one site (74.8% to 79.3%; p<0.0001). There were no significant changes in the proportion of trials with multiple arms (p = 0.22), trials using randomization (p = 0.071), and trials studying pediatric populations (p = 0.20). As would be expected, significantly more trials were ongoing in the late period compared to the early period (50.3% vs 3.7%; p<0.0001). However, even when trial status was assessed for each period with comparable cutoff points (i.e. early– 2012, late– 2018), a smaller proportion of trials in the early period were ongoing compared to the late period (46.7% vs 50.3%; p = 0.006).

**Table 2:** Characteristics of mental health clinical trials registered in ClinicalTrials.gov from October 1, 2007 to April 30, 2018 stratified by early (2007–2012) and late (2013–2018) time periods.

Clinical Trial Characteristics	Time Period		p-value
	Early 2007–2012 n (%)	Late 2013–2018 n (%)	
**Primary Objective**	n = 2,743	n = 3,427	
Treatment	2130 (77.7)	2229 (65.0)	<0.0001
Basic Science	123 (4.5)	252 (7.4)	
Prevention	203 (7.4)	358 (10.4)	
Other	287 (10.5)	588 (17.2)	
**Trial Phases**	n = 2,840	n = 3,462	
Phase 1	311 (11.0)	306 (8.8)	<0.0001
Phase 1/2–2	656 (23.1)	486 (14.0)	
Phase 2/3–3	323 (11.4)	226 (6.5)	
Phase 4	369 (13.0)	263 (7.6)	
Not Applicable	1181 (41.6)	2181 (63.0)	
**Number of arms**	n = 2,759	n = 3,443	
One	415 (15.0)	542 (15.7)	0.22
Two	1758 (63.7)	2230 (64.8)	
≥Three	586 (21.2)	671 (19.5)	
**Funder**	n = 2,840	n = 3,462	
Industry	610 (21.5)	499 (14.4)	<0.0001
AMC/Hosp/Oth	998 (35.1)	1865 (53.9)	
US Govt	1232 (43.4)	1098 (31.7)	
**Blinding**	n = 2,813	n = 3,454	
Double	1014 (36.0)	928 (26.9)	<0.0001
Single	688 (24.5)	1036 (30.0)	
None	1111 (39.5)	1490 (43.1)	
**Randomization**	n = 2,809	n = 3,455	
Randomized	2273 (80.9)	2731 (79.0)	0.071
Non-Randomized	536 (19.1)	724 (21.0)	
**DMC**	n = 2,719	n = 3,261	
Yes	1324 (48.7)	1346 (41.3)	<0.0001
No	1395 (51.3)	1915 (58.7)	
**Studies Children**	n = 2,840	n = 3,462	
Yes	446 (15.7)	586 (16.9)	0.20
No	2394 (84.3)	2876 (83.1)	
**Number of Sites**	n = 2,840	n = 3,462	
One	2124 (74.8)	2745 (79.3)	<0.0001
Two	291 (10.2)	325 (9.4)	
Three-Ten	236 (8.3)	227 (6.6)	
>Ten	189 (6.7)	165 (4.8)	
**Study Status**	n = 2,840	n = 3,462	
Complete	2271 (80.0)	1362 (39.3)	<0.0001
Ongoing	105 (3.7)	1740 (50.3)	
Stopped Early	286 (10.1)	243 (7.0)	
Unknown	178 (6.3)	117 (3.4)	

‘AMC/Hosp/Oth’ denotes Academic Medical Centers/Hospitals/Other. ‘US Govt’ denotes United States Government, which includes NIH (National Institutes of Health) and US Federal agency funders. DMC denotes oversight by a data monitoring committee. Of note, the total number of trials varies slightly by category, as approximately 5% of trials had missing dimensions.

### Mental health trial characteristics compared to non-mental health trials

United States mental health (MH) and non-mental health (NMH) trials in the registry differed in most characteristics (Table 3). A greater proportion of mental health trials noted primary objective as treatment (MH 70.6% vs NMH 64.8%; p<0.0001), were later phase or phase was deemed not applicable (Phase 2/3–3: MH 8.7% vs NMH 6.9%; Phase 4: MH 10.0% vs NMH 7.9%; Not applicable MH 53.3% vs NMH 40.8%; all p<0.0001), and were funded by the United States government (MH 37.0% vs NMH 18.1%; p<0.0001) than non-mental health trials. A greater proportion of mental health trials were also more likely to be conducted at one site (MH 77.3% vs NMH 71.1%; p<0.0001), to have multiple arms (MH 84.6% vs NMH 67.9%; p<0.0001), and to use blinding (MH 58.5% vs NMH 36.9%; p<0.0001), randomization (MH 79.9% vs NMH 58.3%; p<0.0001), and DMCs (MH 44.6% vs NMH 41.3%; p<0.0001). A greater proportion of non-mental health trials were funded by industry (NMH 39.7% vs MH 17.6%; p<0.0001) and were discontinued (NMH 13.1% vs MH 8.4%; p<0.0001).

**Table 3:** Characteristics of mental health and non-mental health clinical trials registered in ClinicalTrials.gov from October 1, 2007 to April 30, 2018.

Clinical Trial Characteristics	Mental Health n (%)	Non-Mental Health n (%)	p-value
**Primary Objective**	n = 6,170	n = 52,955	
Treatment	4359 (70.6)	34299 (64.8)	<0.0001
Basic Science	375 (6.1)	3254 (6.1)	
Prevention	561 (9.1)	5450 (10.3)	
Other	875 (14.2)	9952 (18.8)	
**Trial Phases**	n = 6,302	n = 55,231	
Phase 1	617 (9.8)	11259 (20.4)	<0.0001
Phase 1/2–2	1142 (18.1)	13242 (24.0)	
Phase 2/3–3	549 (8.7)	3805 (6.9)	
Phase 4	632 (10.0)	4374 (7.9)	
Not Applicable	3362 (53.3)	22551 (40.8)	
**Number of arms**	n = 6,202	n = 53,885	
One	957 (15.4)	17289 (32.1)	<0.0001
Two	3988 (64.3)	26149 (48.5)	
≥Three	1257 (20.3)	10447 (19.4)	
**Funder**	n = 6,302	n = 55,231	
Industry	1109 (17.6)	21933 (39.7)	<0.0001
AMC/Hosp/Oth	2863 (45.4)	23293 (42.2)	
US Govt	2330 (37.0)	10005 (18.1)	
**Year Registered**	n = 6,302	n = 55,231	
2007–2012	2840 (45.1)	25205 (45.6)	0.40
2013–2018	3462 (54.9)	30026 (54.4)	
**Blinding**	n = 6,267	n = 54,988	
Double	1942 (31.0)	11791 (21.4)	<0.0001
Single	1724 (27.5)	8484 (15.4)	
None	2601 (41.5)	34713 (63.1)	
**Randomization**	n = 6,264	n = 54,409	
Randomized	5004 (79.9)	31718 (58.3)	<0.0001
Non-Randomized	1260 (20.1)	22691 (41.7)	
**DMC**	n = 5,980	n = 50,730	
Yes	2670 (44.6)	20962 (41.3)	<0.0001
No	3310 (55.4)	29768 (58.7)	
**Number of Sites**	n = 6,302	n = 55,231	
One	4869 (77.3)	39249 (71.1)	<0.0001
Two	616 (9.8)	4731 (8.6)	
Three-Ten	463 (7.3)	6840 (12.4)	
>Ten	354 (5.6)	4411 (8.0)	
**Study Status**	n = 6,302	n = 55,231	
Complete	3633 (57.6)	28526 (51.6)	<0.0001
Ongoing	1845 (29.3)	16945 (30.7)	
Stopped Early	529 (8.4)	7232 (13.1)	
Unknown	295 (4.7)	2528 (4.6)	

‘AMC/Hosp/Oth’ denotes Academic Medical Centers/Hospitals/Other. ‘US Govt’ denotes United States Government, which includes NIH (National Institutes of Health) and US Federal agency funders. DMC denotes oversight by a data monitoring committee. Of note, the total number of trials varies slightly by category, as approximately 5% of trials had missing dimensions.

### Disorders and interventions studied in mental health trials

**Fig 3:**
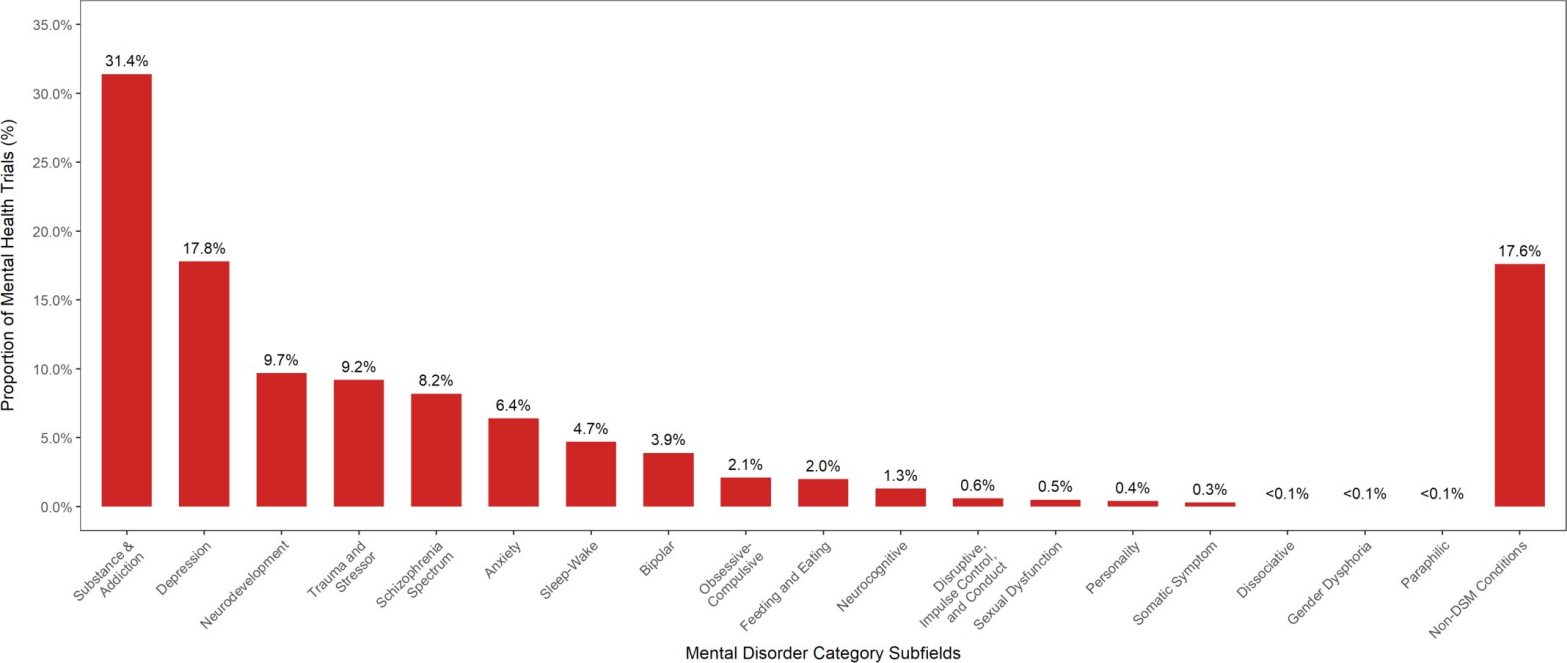
The proportion of total United States mental health clinical trials registered in ClinicalTrials.gov stratified by *DSM-5* disorder categories. These disorder categories correspond to the disorder index categories in the Section II Diagnostic Criteria and Codes provided by the *DSM-5*. Refer to S2 Table for a description of the disorders included under each disorder category. Of note, the total percentage of trials in the ‘Disorder’ category is greater than 100%, as some trials studied more than one disorder category and were counted in each category.

Among registered mental health trials, 5,205 trials (82.6%) focused on six DSM clinical areas: substance use, depression, neurodevelopmental, trauma, schizophrenia spectrum, and anxiety disorders (Fig 3; Table 4). Over the early and late periods, the proportion of trials studying substance use (33.5% to 29.6%, p = 0.00087) and bipolar (4.7% to 3.2%, p = 0.002) disorders decreased, while the proportional of trials studying anxiety (5.3% to 7.3%, p = 0.0021) and neurocognitive (0.7% to 1.8%; p = 0.00023) disorders grew. The proportion of trials studying Non-DSM conditions increased most significantly (13.2% to 21.1%; p<0.0001) and comprised 17.6% of all US mental health trials.

**Table 4:** Disorders and interventions studied in mental health clinical trials registered in ClinicalTrials.gov from October 1, 2007 to April 30, 2018.

	Total	Time Period			Funder			
		Early 2007–2012	Late 2013–2018	P-value	Industry	AMC/Hosp/Oth	US Govt	P-value
	n (%)	n (%)	n (%)		n (%)	n (%)	n (%)	
**Disorder Category**	n = 6,302	n = 2,840	n = 3,462		n = 1,109	n = 2,863	n = 2,330	
Substance	1976 (31.4)	952 (33.5)	1024 (29.6)	0.00087	214 (19.3)	765 (26.7)	997 (42.8)	<0.0001
Depression	1122 (17.8)	522 (18.4)	600 (17.3)	0.29	228 (20.6)	548 (19.1)	346 (14.8)	<0.0001
Non-DSM	1108 (17.6)	376 (13.2)	732 (21.1)	<0.0001	112 (10.1)	639 (22.3)	357 (15.3)	<0.0001
Neurodevelopment	611 (9.7)	273 (9.6)	338 (9.8)	0.87	175 (15.8)	313 (10.9)	123 (5.3)	<0.0001
Trauma	578 (9.2)	283 (10)	295 (8.5)	0.053	34 (3.1)	184 (6.4)	360 (15.5)	<0.0001
Schizophrenia	516 (8.2)	244 (8.6)	272 (7.9)	0.31	190 (17.1)	182 (6.4)	144 (6.2)	<0.0001
Anxiety	402 (6.4)	151 (5.3)	251 (7.3)	0.0021	61 (5.5)	228 (8.0)	113 (4.8)	<0.0001
Sleep	295 (4.7)	140 (4.9)	155 (4.5)	0.43	82 (7.4)	113 (3.9)	100 (4.3)	<0.0001
Bipolar	244 (3.9)	134 (4.7)	110 (3.2)	0.002	52 (4.7)	125 (4.4)	67 (2.9)	0.0065
OCD	131 (2.1)	57 (2.0)	74 (2.1)	0.79	22 (2.0)	84 (2.9)	25 (1.1)	0.00017
Feeding	127 (2)	61 (2.1)	66 (1.9)	0.56	20 (1.8)	62 (2.2)	45 (1.9)	0.72
Neurocognitive	85 (1.3)	21 (0.7)	64 (1.8)	0.00023	3 (0.3)	65 (2.3)	17 (0.7)	<0.0001
Disruptive	36 (0.6)	14 (0.5)	22 (0.6)	0.56	2 (0.2)	20 (0.7)	14 (0.6)	0.15
Sexual	31 (0.5)	12 (0.4)	19 (0.5)	0.59	14 (1.3)	15 (0.5)	2 (0.1)	<0.0001
Personality	26 (0.4)	12 (0.4)	14 (0.4)	1.0	3 (0.3)	17 (0.6)	6 (0.3)	0.12
Somatic	16 (0.3)	3 (0.1)	13 (0.4)	0.062	0 (0)	10 (0.3)	6 (0.3)	0.15
Movement	5 (0.1)	1 (0)	4 (0.1)	0.49	3 (0.3)	2 (0.1)	0 (0)	0.03
Dissociative	2 (0)	1 (0)	1 (0)	1.0	1 (0.1)	1 (0)	0 (0)	0.38
Gender	2 (0)	0 (0)	2 (0.1)	0.57	0 (0)	0 (0)	2 (0.1)	0.18
Paraphilic	2 (0)	0 (0)	2 (0.1)	0.57	0 (0)	0 (0)	2 (0.1)	0.18
**Intervention**	n = 6,302	n = 2,840	n = 3,462		n = 1,109	n = 2,863	n = 2,330	
Behavioral	2489 (39.5)	1008 (35.5)	1481 (42.8)	<0.0001	71 (6.4)	1249 (43.6)	1169 (50.2)	<0.0001
Pharmaceutical (Pharm)	2189 (34.7)	1234 (43.5)	955 (27.6)	<0.0001	849 (76.6)	792 (27.7)	548 (23.5)	<0.0001
Other (Oth)	803 (12.7)	254 (8.9)	549 (15.9)	<0.0001	108 (9.7)	455 (15.9)	240 (10.3)	<0.0001
Beh + Pharm	254 (4.0)	125 (4.4)	129 (3.7)	0.2	18 (1.6)	114 (4.0)	122 (5.2)	<0.0001
Beh + Oth	240 (3.8)	85 (3.0)	155 (4.5)	0.0027	7 (0.6)	104 (3.6)	129 (5.5)	<0.0001
Pharm + Oth	264 (4.2)	109 (3.8)	155 (4.5)	0.23	49 (4.4)	127 (4.4)	88 (3.8)	0.46
Beh + Pharm + Oth	63 (1.0)	25 (0.9)	38 (1.1)	0.46	7 (0.6)	22 (0.8)	34 (1.5)	0.018

Total number of trials by disorder category and interventions studied is included in the left-most column. The data are stratified by period of submission (early and late) and by funder (Industry, AMC/Hosp/Oth, and US Govt). ‘AMC/Hosp/Oth’ denotes Academic Medical Centers/Hospitals/Other. ‘US Govt’ denotes United States Government, which includes NIH (National Institutes of Health) and US Federal agency funders. Refer to S2 Table for a description of the disorders included under each disorder category. Of note, the total percentage of trials in the ‘Disorder’ category is greater than 100%, as some trials studied more than one disorder category and were counted in each category. For intervention types, ‘Beh’ denotes Behavioral, ‘Pharm’ denotes pharmaceutical, and ‘Oth’ denotes Other. See methods for the interventions included in each of these categories. Trials in which multiple intervention types were tested are labeled with all relevant interventions (e.g. a trial testing psychotherapy and pharmacotherapy was labeled ‘Beh + Pharm’).

The proportion of trials studying each disorder category also differed significantly by funder (Table 4). A larger proportion of trials funded by industry studied depression (Ind 20.6%, AMC/Hosp/Oth 19.1%, US Govt 14.8%; p<0.0001), neurodevelopmental (Ind 15.8%, AMC/Hosp/Oth 10.9%, US Govt 5.3%; p<0.0001), schizophrenia spectrum (Ind 17.1%, AMC/Hosp/Oth 6.4%, US Govt 6.2%; p<0.0001), sleep (Ind 7.4%, AMC/Hosp/Oth 3.9%, US Govt 4.3%; p<0.0001), and sexual (Ind 1.3%, AMC/Hosp/Oth 0.5%, Us Govt 0.1%; p<0.0001) disorders compared to academic medical center/hospital/other and the US government funders. A larger proportion of trials funded by academic medical centers, hospitals and other funders studied Non-DSM (AMC/Hosp/Oth 22.3%, Ind 10.1%, US Govt 15.3%; p<0.0001), anxiety (AMC/Hosp/Oth 8.0%, Ind 5.5%, US Govt 4.8%; p<0.0001), and OCD (AMC/Hosp/Oth 2.9%, Ind 2.0%, US Govt 1.1%; p = 0.00017) disorders compared to industry and US government funders. A larger proportion of US government-funded trials studied substance use (US Govt 42.8%, Ind 19.3%, AMC/Hosp/Oth 26.7%; p<0.0001) and trauma (US Govt 15.5%, Ind 3.1%, AMC/Hosp/Oth 6.4%; p<0.0001) disorders compared to industry and academic medical center/hospital/other funders.

The proportion of trials studying each intervention type differed by time period and funders as well (Table 4). Over the early and late time periods, the proportion of trials that studied behavioral (35.5% to 42.8%; p<0.0001) or Other interventions (8.9% to 15.9%; p<0.0001) grew significantly, as did trials studying both behavioral and Other interventions (3.0% to 4.5%; p = 0.0027), whereas the proportion of trials studying pharmaceutical interventions (43.5% to 27.6%; P<0.0001) decreased. US government funders studied a larger proportion of behavioral interventions (US Govt 50.2%, Ind 6.4%, AMC/Hosp/Oth 43.6%; p<0.0001), behavioral interventions tested alongside pharmaceuticals (US Govt 5.2%, Ind 1.6%, AMC/Hosp/Oth 4.0%; p<0.0001), and behavioral interventions tested alongside Other interventions (Us Govt 5.5%, Ind 0.6%, AMC/Hosp/Oth 3.6%; p<0.0001) than industry or academic medical center/hospital/other funders. Industry funders studied a larger proportion of pharmaceutical interventions (Ind 76.6%, AMC/Hosp/Oth 27.7%, US Govt 23.5%; p<0.0001) than academic medical center/hospital/other and US government funders, and academic medical center/hospital/other funders studied a larger proportion of Other interventions (AMC/Hosp/Oth 15.9%, Ind 9.7%, US Govt 10.3%; p<0.0001) than either of the other two funder categories.

### Mental health trial sponsorship

The fifty organizations most commonly reported as the sponsor for trials accounted for 53.2% (3,355/6,302) of all US mental health interventional trials in our sample (Table 5). Forty-two (84%) were academic institutions or hospitals, four were pharmaceutical companies, and four were US governmental agencies (though two were sponsor names under the larger umbrella of the United States Veterans Association). When compared by time period (i.e. early [2007–2012] and late [2013–2018]), the number of trials sponsored by three of the top industry sponsors and three of the top US government agencies decreased, whereas the number of trials sponsored by thirty-four of the forty-two top academic medical center/hospital/other funders (81.0%) increased.

**Table 5:** Top fifty sponsors of United States mental health clinical trials registered in ClinicalTrials.gov from October 1, 2007 to April 30, 2018.

Sponsor	Sponsor Type	Number of Trials		
		2007–2012	2013–2018	Total
Yale University	AMC/Hosp/Oth	130	129	259
Massachusetts General Hospital	AMC/Hosp/Oth	127	96	223
VA Office of Research and Development	US Govt	73	137	210
New York State Psychiatric Institute	AMC/Hosp/Oth	73	97	170
University of California, Los Angeles	AMC/Hosp/Oth	62	54	116
University of Pennsylvania	AMC/Hosp/Oth	58	51	109
University of California, San Francisco	AMC/Hosp/Oth	47	60	107
Johns Hopkins University	AMC/Hosp/Oth	49	51	100
Medical University of South Carolina	AMC/Hosp/Oth	40	58	98
Duke University	AMC/Hosp/Oth	37	53	90
Stanford University	AMC/Hosp/Oth	41	48	89
University of Pittsburgh	AMC/Hosp/Oth	40	41	81
University of North Carolina, Chapel Hill	AMC/Hosp/Oth	32	44	76
Mayo Clinic	AMC/Hosp/Oth	37	35	72
University of Washington	AMC/Hosp/Oth	33	38	71
University of Michigan	AMC/Hosp/Oth	20	49	69
University of Minnesota—Clinical and Translational Science Institute	AMC/Hosp/Oth	26	40	66
McLean Hospital	AMC/Hosp/Oth	38	27	65
Emory University	AMC/Hosp/Oth	13	47	60
New York University School of Medicine	AMC/Hosp/Oth	10	48	58
University of California, San Diego	AMC/Hosp/Oth	27	30	57
Weill Medical College of Cornell University	AMC/Hosp/Oth	27	29	56
University of Wisconsin, Madison	AMC/Hosp/Oth	26	28	54
Brown University	AMC/Hosp/Oth	26	27	53
University of Maryland	AMC/Hosp/Oth	24	28	52
Pfizer	Industry	42	7	49
US Department of Veterans Affairs	US Govt	45	1	46
Washington University School of Medicine	AMC/Hosp/Oth	18	25	43
Dartmouth-Hitchcock Medical Center	AMC/Hosp/Oth	15	27	42
UConn Health	AMC/Hosp/Oth	30	12	42
University of Texas at Austin	AMC/Hosp/Oth	7	35	42
Indiana University	AMC/Hosp/Oth	17	24	41
Butler Hospital	AMC/Hosp/Oth	14	26	40
Baylor College of Medicine	AMC/Hosp/Oth	23	16	39
Northwestern University	AMC/Hosp/Oth	9	30	39
National Institute on Drug Abuse (NIDA)	US Govt	25	13	38
Icahn School of Medicine at Mount Sinai	AMC/Hosp/Oth	15	22	37
University of Colorado, Denver	AMC/Hosp/Oth	10	23	33
University of Texas Southwestern Medical Center	AMC/Hosp/Oth	16	17	33
Children's Hospital Medical Center, Cincinnati	AMC/Hosp/Oth	17	15	32
The University of Texas Health Science Center, Houston	AMC/Hosp/Oth	10	22	32
National Institute on Alcohol Abuse and Alcoholism (NIAAA)	US Govt	18	13	31
University of Cincinnati	AMC/Hosp/Oth	15	16	31
University of Miami	AMC/Hosp/Oth	11	20	31
Sunovion	Industry	14	16	30
Forest Laboratories	Industry	24	5	29
Shire	Industry	18	11	29
University of Florida	AMC/Hosp/Oth	5	24	29
Boston Medical Center	AMC/Hosp/Oth	9	19	28
University of California, Davis	AMC/Hosp/Oth	9	19	28

The number of trials sponsored by each agency stratified by time period of submission (i.e. early [2007–2012] and late [2013–2018]) and assessed in total. ‘AMC/Hosp/Oth’ denotes Academic Medical Centers/Hospitals/Other. ‘US Govt’ denotes United States Government, which includes NIH (National Institutes of Health) and US Federal agency sponsors.

### Mental health trial discontinuation

**Fig 4:**
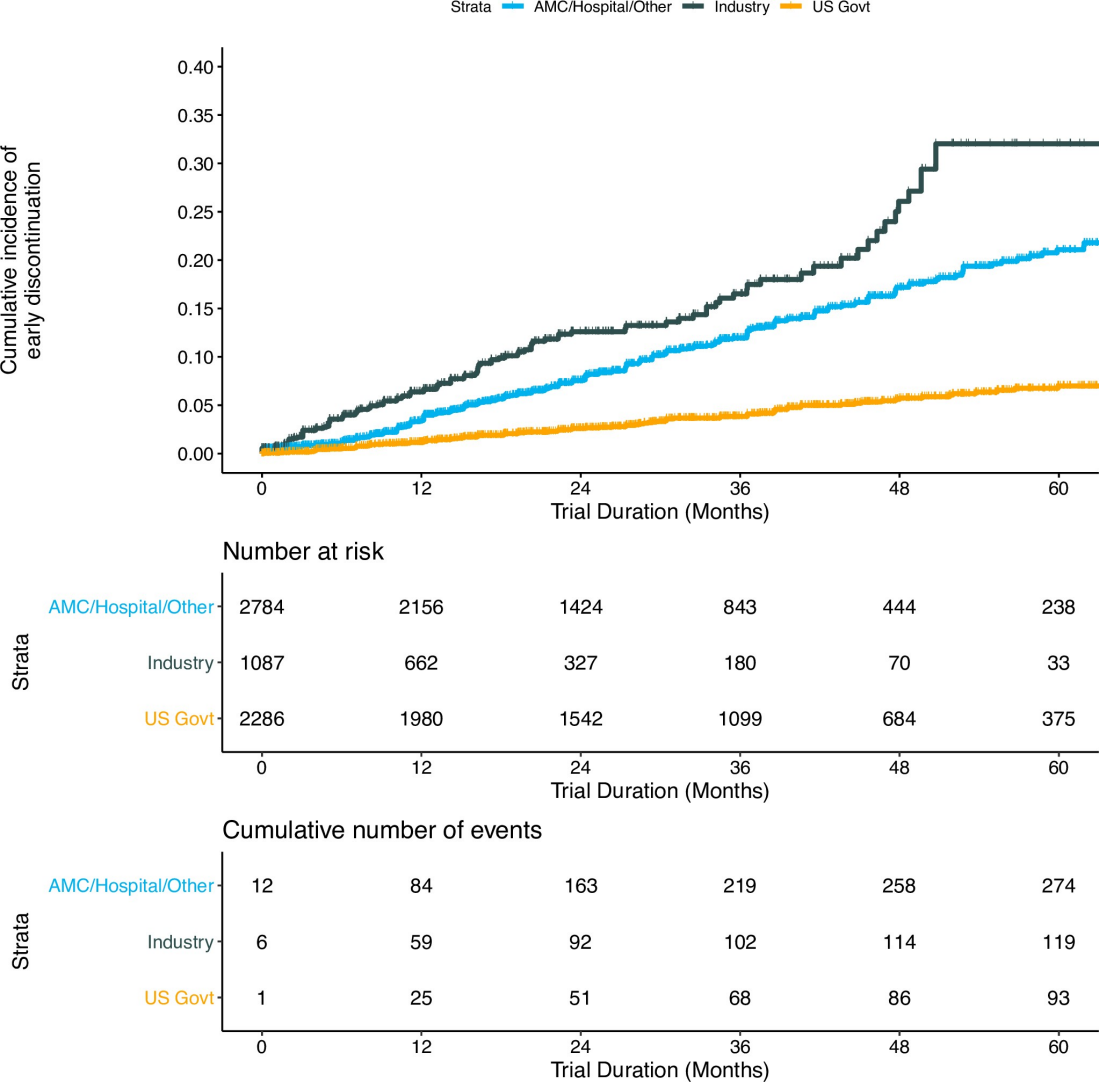
Cumulative incidence of discontinuation among United States mental health clinical trials. Measured from time after the trial start date. Stratified by funder type. AMC/Hosp/Oth’ denotes Academic Medical Centers/Hospitals/Other. ‘US Govt’ denotes United States Government, which includes NIH (National Institutes of Health) and US Federal agency funders.

A total of 529 trials (8.4% of US mental health trials in our sample), representing an actual enrollment of 18,226 participants, were discontinued. Of the discontinued trials, 331 were terminated after enrollment began, 31 were suspended, and 167 were withdrawn before participant recruitment. Industry funders had the largest proportion of discontinued trials (Ind 11.6%, AMC/Hosp/Oth 10.3%, US Govt 4.5%; p<0.0001). Multivariate regression analysis revealed that intervention studied, funder type, and oversight by a DMC were all related to trial discontinuation (Table 6). Trials studying pharmaceuticals (adjusted hazard ratio [aHR] 2.44, 95% confidence interval [CI] 1.73–3.45; p<0.0001) and pharmaceuticals with Other interventions (aHR 3.65, 95% CI 2.37–5.60; p<0.0001) were more likely to be discontinued compared to trials studying behavioral interventions. Trials funded by industry (aHR 2.86, 95% CI 2.07–3.96; p<0.0001) and academic medical center/hospital/other funders (aHR 2.59, 95% CI 2.01–3.35; p<0.0001) were more likely than trials funded by US government agencies to be discontinued. Trials with oversight from data monitoring committees (DMC) were less likely to be discontinued compared to trials without DMCs (aHR 0.64, 95% CI 0.53–0.78; p<0.0001). Kaplan-Meier curves (Fig 4) show cumulative incidence of trial discontinuation within the first five years of the trial start date, stratified by funder. Throughout this period, industry-funded trials had the highest rates of discontinuation, with US government-funded trials demonstrating the lowest rate of discontinuation.

**Table 6:** Cox proportional hazards regression analysis of factors associated with United States mental health trial discontinuation.

	Univariable		Multivariable	
n = 5,818	HR (95% CI)	p-value	aHR (95% CI)	p-value
**Primary Objective**				
Treatment	Reference		Reference	
Basic Science	0.74 (0.47–1.18)	0.21	0.63 (0.39–1.01)	0.058
Other	0.86 (0.65–1.14)	0.29	1.02 (0.76–1.36)	0.92
Prevention	0.66 (0.46–0.94)	0.021	0.97 (0.65–1.43)	0.87
**Intervention**				
Behavioral (Beh)	Reference		Reference	
Pharmaceutical (Pharm)	3.47 (2.75–4.36)	<0.0001	2.44 (1.73–3.45)	<0.0001
Other (Oth)	1.84 (1.30–2.60)	0.00058	1.42 (0.99–2.04)	0.059
Beh + Pharm	1.42 (0.84–2.41)	0.19	1.34 (0.76–2.34)	0.31
Beh + Oth	1.30 (0.71–2.36)	0.40	1.59 (0.87–2.90)	0.13
Pharm + Oth	4.49 (3.10–6.50)	<0.0001	3.65 (2.37–5.60)	<0.0001
Beh + Pharm + Oth	0.00 (0.00 - >100)	0.99	0.00 (0.00 - >100)	0.99
**Trial Phase**				
Not Applicable	Reference		Reference	
Phase 1	1.84 (1.32–2.57)	0.00034	1.31 (0.92–1.88)	0.14
Phase 1/2–2	1.78 (1.40–2.27)	<0.0001	1.27 (0.94–1.71)	0.10
Phase 2/3–3	1.92 (1.42–2.60)	<0.0001	1.23 (0.86–1.75)	0.26
Phase 4	2.47 (1.91–3.17)	<0.0001	1.18 (0.86–1.61)	0.30
**Year Registered**				
2007–2012	Reference		Reference	
2013–2018	1.17 (0.97–1.40)	0.10	1.17 (0.97–1.42)	0.10
**Funder**				
US Govt	Reference		Reference	
Industry	5.38 (4.05–7.15)	<0.0001	2.86 (2.07–3.96)	<0.0001
AMC/Hosp/Oth	3.40 (2.67–4.34)	<0.0001	2.59 (2.01–3.35)	<0.0001
**Blinding**				
None	Reference		Reference	
Single	0.43 (0.33–0.57)	<0.0001	0.58 (0.42–0.80)	0.0011
Double	1.44 (1.18–1.75)	0.00026	0.95 (0.69–1.29)	0.73
**Randomization**				
Non-Randomized	Reference		Reference	
Randomized	0.63 (0.51–0.78)	<0.0001	0.96 (0.71–1.31)	0.81
**Reports DMC**				
No	Reference		Reference	
Yes	0.58 (0.48–0.70)	<0.0001	0.64 (0.53–0.78)	<0.0001
**Studies Children**				
No	Reference		Reference	
Yes	0.90 (0.71–1.16)	0.42	1.18 (0.89–1.56)	0.26
**Number of Sites**				
One	Reference		Reference	
≥Two	0.78 (0.62–0.99)	0.038	0.74 (0.58–0.95)	0.019
**Disorder Category**				
Substance	0.55 (0.44–0.69)	<0.0001	0.67 (0.49–0.92)	0.013
Depression	1.23 (0.99–1.54)	0.062	0.99 (0.74–1.32)	0.96
Schizophrenia	1.51 (1.14–2.01)	0.0042	1.04 (0.73–1.48)	0.84
Neurodevelopment	0.99 (0.72–1.36)	0.96	0.61 (0.41–0.92)	0.017
Anxiety	1.48 (1.07–2.04)	0.019	1.27 (0.89–1.82)	0.19
Trauma	0.88 (0.65–1.20)	0.43	1.29 (0.89–1.86)	0.18
Sleep	1.29 (0.86–1.93)	0.21	1.04 (0.67–1.62)	0.85
Bipolar	1.39 (0.96–2.00)	0.078	0.90 (0.60–1.35)	0.62
OCD	1.07 (0.59–1.94)	0.83	0.73 (0.38–1.40)	0.34
Feeding	0.88 (0.45–1.70)	0.70	0.81 (0.40–1.63)	0.56
Neurocognitive	2.55 (1.53–4.27)	0.00036	1.77 (1.00–3.15)	0.051
Non-DSM	0.92 (0.71–1.18)	0.50	0.89 (0.67–1.20)	0.45

All listed variables were included in the multivariable regression. ‘HR’ denotes hazard ratio, and ‘aHR’ denotes adjusted hazard ratio. ‘95% CI’ denotes 95% confidence interval. ‘DMC’ denotes Data Monitoring Committee. ‘AMC/Hosp/Oth’ denotes Academic Medical Centers/Hospitals/Other. ‘US Govt’ denotes United States Government, which includes NIH (National Institutes of Health) and US Federal agency funders. Refer to S2 Table for a description of the disorders included under each disorder category. For intervention types, ‘Beh’ denotes Behavioral, ‘Pharm’ denotes pharmaceuticals, and ‘Oth’ denotes Other. See methods for the interventions included in each of these categories. Trials in which multiple intervention types were tested are labeled with all relevant interventions (e.g. a trial testing psychotherapy and pharmacotherapy was labeled ‘Beh + Pharm’).

### Results reporting of mental health trials to ClinicalTrials.gov

Of the 2,197 trials completed by April 30, 2015, 930 (42.3%) reported results to the registry, and only 644 (29.3%) reported results to the registry within 36 months of completion (i.e. by April 30, 2018), which was the maximum time allowed for certain trials by the FDAAA. In multivariate regression analysis, intervention, trial phase, funder, and disorder category were all associated with results reporting (Table 7). Trials studying pharmaceutical or Other interventions were more likely to report results than trials studying behavioral interventions (Pharm adjusted odds ratio [aOR] 7.47, 95% CI 5.05–11.03; Oth aOR 3.16 95% CI 2.12–4.72; both p<0.0001). Phase 1 trials were less likely to report results than other phase trials or trials in which phase designation was not applicable (Phase 1 aOR 0.25, 95% CI 0.16–0.39, p<0.0001). Trials funded by industry or academic medical center/hospital/other funders were less likely to report results than US government funders (Ind aOR 0.70, 95% CI 0.50–0.97, p = 0.034; AMC/Hosp/Oth aOR 0.73, 95% CI 0.56–0.96, p = 0.022). Many disorder categories were more likely to report results than the least studied disorders, which included disruptive, sexual, personality, somatic, movement, dissociative, gender, and paraphilic disorders. Disorder categories with the most significant results reporting included substance use (aOR 1.97, 95% CI 1.38–2.82, p = 0.00021), trauma (aOR 3.19, 95% CI 2.06–4.96, p<0.0001), OCD (aOR 5.76, 95% CI 2.50–13.28, p<0.0001), and Non-DSM conditions (aOR 1.75, 95% CI 1.19–2.57, p = 0.0042). Of note, a separate regression of results reporting to the registry at any time (i.e. not just restricted to the 36-month window maximally allowed for some trials by the FDAAA) showed no significant differences to the above findings.

**Table 7:** Logistic regression analysis of United States mental health trial characteristics associated with reporting results to ClinicalTrials.gov within 36 months of trial completion.

	Univariable		Multivariable	
n = 2,197	OR (95% CI)	p-value	aOR (95% CI)	p-value
**Primary Objective**				
Treatment	Reference		Reference	
Basic Science	0.76 (0.49–1.19)	0.23	0.72 (0.43–1.19)	0.20
Other	0.54 (0.38–0.76)	0.00050	0.68 (0.46–1.00)	0.047
Prevention	0.34 (0.21–0.54)	<0.0001	0.71 (0.42–1.19)	0.20
**Intervention**				
Behavioral (Beh)	Reference		Reference	
Pharmaceutical (Pharm)	4.95 (3.86–6.36)	<0.0001	7.47 (5.05–11.03)	<0.0001
Other (Oth)	2.64 (1.82–3.83)	<0.0001	3.16 (2.12–4.72)	<0.0001
Beh + Pharm	8.31 (5.18–13.32)	<0.0001	9.44 (5.61–15.88)	<0.0001
Beh + Oth	1.14 (0.54–2.38)	0.73	1.10 (0.51–2.38)	0.80
Pharm + Oth	3.99 (2.34–6.79)	<0.0001	5.16 (2.78–9.60)	<0.0001
Beh + Pharm + Oth	4.10 (1.55–10.82)	0.0044	5.35 (1.88–15.27)	0.0017
**Trial Phase**				
Not Applicable	Reference		Reference	
Phase 1	0.52 (0.35–0.79)	0.0019	0.25 (0.16–0.39)	<0.0001
Phase 1/2–2	1.68 (1.31–2.15)	<0.0001	0.72 (0.52–0.99)	0.041
Phase 2/3–3	2.18 (1.61–2.94)	<0.0001	1.03 (0.71–1.49)	0.87
Phase 4	3.17 (2.35–4.28)	<0.0001	1.10 (0.76–1.59)	0.62
**Year Registered**				
2007–2012	Reference		Reference	
2013–2015	0.64 (0.48–0.85)	0.0025	0.82 (0.59–1.14)	0.24
**Funder**				
US Govt	Reference		Reference	
Industry	1.39 (1.09–1.75)	0.0068	0.70 (0.50–0.97)	0.034
AMC/Hosp/Oth	0.80 (0.64–1.01)	0.058	0.73 (0.56–0.96)	0.022
**Blinding**				
None	Reference		Reference	
Single	0.91 (0.70–1.17)	0.46	1.14 (0.81–1.59)	0.46
Double	2.16 (1.74–2.69)	<0.0001	0.96 (0.67–1.38)	0.82
**Randomization**				
Non-Randomized	Reference		Reference	
Randomized	0.96 (0.76–1.22)	0.75	1.00 (0.69–1.44)	0.98
**Reports DMC**				
No	Reference		Reference	
Yes	1.28 (1.06–1.55)	0.011	1.16 (0.93–1.45)	0.18
**Studies Children**				
No	Reference		Reference	
Yes	0.64 (0.48–0.84)	0.0017	0.74 (0.51–1.07)	0.11
**Number of Sites**				
One	Reference		Reference	
≥Two	1.40 (1.13–1.73)	0.0019	1.23 (0.96–1.59)	0.11
**Disorder Category**				
Substance	1.23 (1.01–1.50)	0.043	1.97 (1.38–2.82)	0.00021
Depression	0.90 (0.70–1.15)	0.39	1.45 (1.01–2.07)	0.045
Schizophrenia	1.05 (0.75–1.47)	0.76	1.41 (0.90–2.21)	0.13
Neurodevelopment	1.15 (0.85–1.57)	0.36	1.75 (1.10–2.79)	0.019
Anxiety	0.86 (0.56–1.32)	0.48	1.33 (0.79–2.22)	0.28
Trauma	1.23 (0.89–1.71)	0.20	3.19 (2.06–4.96)	<0.0001
Sleep	0.69 (0.43–1.13)	0.14	0.81 (0.47–1.41)	0.46
Bipolar	1.74 (1.14–2.67)	0.010	1.71 (1.02–2.88)	0.044
OCD	2.34 (1.19–4.62)	0.014	5.76 (2.50–13.28)	<0.0001
Feeding	0.65 (0.30–1.44)	0.29	1.05 (0.41–2.70)	0.91
Neurocognitive	1.02 (0.31–3.34)	0.97	1.06 (0.30–3.78)	0.93
Non-DSM	0.83 (0.62–1.10)	0.19	1.75 (1.19–2.57)	0.0042

All listed variables were included in the multivariable regression. Data are for trials completed on or before April 30, 2015 (n = 2,197). ‘OR’ denotes odds ratio and ‘aOR’ denotes adjusted odds ratio. ‘95% CI’ denotes 95% confidence interval. ‘DMC’ denotes Data Monitoring Committee. ‘AMC/Hosp/Oth’ denotes Academic Medical Centers/Hospitals/Other. ‘US Govt’ denotes United States Government, which includes NIH (National Institutes of Health) and US Federal agency funders. Refer to S2 Table for a description of the disorders included under each disorder category. For intervention types, ‘Beh’ denotes Behavioral, ‘Pharm’ denotes pharmaceuticals, and ‘Oth’ denotes Other. Trials in which multiple intervention types were tested are labeled with all relevant interventions (e.g. a trial testing psychotherapy and pharmacotherapy was labeled ‘Beh + Pharm’). See methods for the interventions included in each of these categories.

## Discussion

This study aims to provide clarity to the landscape of contemporary US mental health clinical trials registered in ClinicalTrials.gov. Our analysis helps us to better understand how mental health trial features, including funders, trial design, and disorders and interventions studied, have changed over time, and how many of these trial features differ from non-mental health trials in the registry. This study also provides insight into trial characteristics that may influence or are at least correlated with trial discontinuation and results reporting to the registry.

Mental health trials made up 10.2% of the United States interventional trials registered in ClinicalTrials.gov from October 1, 2007 to April 30, 2018. Industry and US government-funded trials demonstrated a significant annual decline (-9.6% and -3.9%, respectively). This decline was counterbalanced by the 9.2% annual growth of trials funded by academic, hospital, and other funders.

The decline in US government and industry funders in clinical research is in accordance with the literature [27, 28]. Our data suggest that the decline in US government-funded trials is not unique to psychiatry (Mental Health CAGR –3.9% vs Non-Mental Health -2.3%), which is consistent with reports of a 27% reduction in the number of trials funded by the National Institutes of Health across all medical specialties from 2006 to 2014 [29], as well as a 32% decrease in funding ($110 million in 2011 to $75 million in 2014) for the National Institute of Mental Health [28]. Despite these larger trends in depreciating US government funding, the US Department of Veterans Affairs (VA) has significantly expanded its priority to fund psychological research and has nearly tripled the number of mental health professionals it employed since 2006 [30]. We found VA-funded trials increased overall by 17% between the early and late time periods of our study (118 trials [2007–2012] to 138 trials [2013–2018]), and it was the largest funder of mental health trials in the late period. Of note, VA trial sponsors were listed as either ‘US Department of Veterans Affairs’ or ‘VA Office of Research and Development’ in ClinicalTrials.gov during the early period, though the latter sponsor name was used almost exclusively in the late period. We tallied the total number of trials under both names to assess this trend.

The causes of the decrease in industry-funded mental health trials are likely multifactorial and include the increasing cost of developing new drugs, greater time required to bring each drug to market, reduced market exclusivity for new medications, and lower demand for branded drugs by increasingly cost-conscious payers [18, 27]. Multiple international pharmaceutical companies have significantly decreased their investments in new treatments for depression, bipolar disorder, and schizophrenia, and some companies, such as GlaxoSmithKline, have closed their psychiatric units altogether [18, 19]. Our data show that the number of new industry-funded clinical trials in mental health decreased two-times faster than industry-funded non-mental health trials in the registry (Mental Health CAGR -9.6 vs Non-Mental Health CAGR -4.7%). This suggests that, while industry funders are decreasing across all areas of medicine, industry may be specifically repositioning away from mental health research, or it may be devoting more resources to mental health research outside of the United States. While some have suggested that the reduction in industry-funded mental health research is the result of companies partnering with external collaborators, our analysis, which captures such collaborations as industry-funded, suggests otherwise [19].

While philanthropic support has accounted for less than one percent of funding for mental health research historically [31], shrinking funding from US governmental agencies and industry has pushed mental health researchers to pursue charitable giving as an alternative funding source [32]. This funding realignment has required new research strategies within academic institutions and hospitals, which now account for 19 of the top 20 sponsors of mental health trials. It is important to note, however, that ‘funder’ is a self-reported category within ClinicalTrials.gov, and there may have been trials that did receive funding from Industry or US government collaborators but did not report these collaborations. Further analysis of the sources of funding for academic medical center/hospital/other trials could potentially be assessed through use of the Secondary ID Numbers provided in ClinicalTrials.gov, which include grant and other funding information in a free-text field. Additional information is also available in the Protocol Registration ClinicalTrials.gov Data Element Definitions. While these approaches were beyond the scope of this study, they are promising areas of further research to clarify this issue of funding sources for trials other than industry or US government agencies.

Our analysis shows that the disorders and interventions studied by trials differed significantly by funder. As might be expected, the largest proportion of industry trials (76.6%) studied pharmaceutical interventions. Industry-funded trials preferentially studied depression, neurodevelopmental, schizophrenia spectrum, and sleep disorders, all disorders with classes of pharmaceuticals that are mainstays of care for these disorders (i.e. antidepressants, stimulants, antipsychotics, and hypnotics). The US governmental agencies preferentially studied substance use and trauma disorders. This is in keeping with the growth of US Department of Veterans Affairs (VA) funding, as these disorders are prevalent among veterans [33]. Academic/hospitals and US government agencies funded the majority of trials that studied conditions not clearly defined by *DSM-5* diagnostic criteria (i.e. Non-DSM conditions), and trials studying Non-DSM conditions showed the largest growth of any disorder category from 2007 to 2018. This may reflect the efforts of the National Institute of Mental Health and other funding bodies to adopt the Research Domain Criteria (RDoC) in an effort to move away from studying *DSM* diagnoses and towards studying brain systems that often cross traditional diagnostic boundaries [34]. Both US government and academic medical center/hospital/other funders studied a large proportion of behavioral interventions (US Govt 50.2%, AMC/Hosp/Oth 43.6%, and 97.1% of all behavioral trials), as well as trials that compared behavioral interventions to pharmaceuticals or other interventions, such as transracial magnetic stimulation. This is consistent with the growing appreciation within mental health that there are often synergistic effects of psychotherapy and psychopharmacology [35].

Our data demonstrate that trial design features of registered US mental health trials changed overtime in multiple key respects. Mental health trials have increasingly been single or non-blinded and are not monitored by data monitoring committees (DMCs). While at face value these changes seem disappointing, they may rather reflect the growth of behavioral intervention trials, many of which cannot be blinded and do not required DMC oversight. US regulations only require DMCs for trials testing new drugs, biologics, or devices, in double blinded studies where there is considerable risk to patients, or when research is conducted in vulnerable populations (e.g. prisoners) [36].

There are likely many reasons why US mental health (MH) trials differed from non-mental health (NMH) trials, and the heterogeneity of the non-mental health category limits its interpretability. However, it is striking that non-mental health trials were more than twice as likely to be funded by industry (NMH 39.7% vs MH 17.6%), and non-mental health trials were less likely to be blinded (NMH 36.9% vs MH 58.5%), randomized (NMH 58.3% vs MH 79.9%), or monitored by a DMC (NMH 41.3% vs MH 44.6%). In 2018, none of the top ten highest grossing pharmaceutical products were for mental health indications, which may reflect why there is such disparity in industry sponsorship between mental health and non-mental health trials [37]. There are many instances, such as in surgical trials, where blinding and randomization may be infeasible or unethical, which likely accounts for the lower proportion of non-mental health trials using these design features [38]. This is also consistent with a prior comparison of mental health trials to oncology and cardiovascular trials [12]. Perhaps most salient to the comparison between mental health and non-mental health trials, it is reassuring that over our study period (comparing 2007–2012 to 2013–2018), the change in percentage of mental health trials reaching completion and using trial design measures to limit bias has not lagged behind non-mental health studies. Although the percentage of mental health trials utilizing double blinding and DMCs has decreased over time, this trend has also occurred in non-mental health trials (Double blinding: Early period 23.1%, Late period 20.1%, p<0.0001; DMCs: Early period 42.7%, Late period 40.2%, p<0.0001).

Even though fewer US mental health trials were discontinued than their non-mental health counterparts, one in twelve registered mental health clinical trials was stopped early over the 127-month period analyzed. Industry funders had the greatest proportion of discontinued trials, and 18,226 participants were enrolled in eventually discontinued trials. There are many justifiable reasons for trial termination, particularly in pilot studies (comprising 17.4% of discontinued trials in our sample), including poor patient accrual and lack of intervention efficacy, though commercial considerations remain controversial [39]. Industry may have less tolerance for risk and a shorter view of return on investment compared to the US government or academic medical center/hospital/other funders [19]. The increasing competitiveness of US government-allotted funding may also select for more rigorously designed and feasible trials than those funded by industry [28]. Together, this suggests that industry funders may have fewer restrictions for initiating trials but also a lower bar for stopping a trial early based on initial findings.

We found that 57.7% of completed trials did not report results to the registry, and only 29.3% reported results within 36 months of completion. This is consistent with prior studies of insufficient registration and results reporting in mental health clinical research [40]. The FDAAA and Final Rule do not mandate that all trials report their results to a registry, which is likely why so few have [6]. Moreover, the Final Rule, which expanded the proportion of trials mandated to report results to a registry, only took effect on January 18, 2017, which is outside the window of the trials studied in our sample. Results reporting has likely increased for trials registered after this date. Early phase trials were significantly less likely to report results, which is consistent with previous analyses using the ClinicalTrial.gov registry [24]. Funding was associated with results reporting, with industry and academic medical center/hospital/other funders less likely to report results to the registry than US government agencies. US government funders may have devoted administrative and research support to comply with reporting, and it is possible that a greater percentage of their trials meet the requirement for mandated reporting by the FDAAA and Final Rule. Intervention type was also associated with results reporting, with trials studying behavioral interventions the least likely to report results to the registry. This is likely because the FDAAA only mandated registration and results reporting for certain trials studying pharmaceuticals or devices, and so there is less incentive for reporting for behavioral trials. It is unfortunate that so few trials report their results to the registry. Dissemination of research findings is crucial for informing clinical practice, and selective reporting can lead to distortion of the field’s knowledge base. Completed trials with inaccessible data represent a poor return for finite research resources, as well as a potential failure to meet the legal and ethical obligation that investigators have to trial participants, including as a component of informed consent.

Our study has multiple limitations. First, while ClinicalTrials.gov is one of the largest international trial registries and contains 70% of trials registered in the International Clinical Trials Registry of the World Health Organization, it is not an exhaustive list of all US clinical trials [14]. Second, not all trials, such as phase 1 trials or trials studying non-pharmacologic interventions, were subject to the FDAAA or the Final Rule requirements [6]. There may be other incentives and norms that bias the registration of trials with certain characteristics, and trends identified in the registry may at least in part reflect changes in trial reporting instead of changes in how trials were conducted or designed. Nevertheless, ClinicalTrials.gov is a unique resource that has allowed many medical specialties to assess features and trends in their clinical research, which might otherwise remain unassessable [12–14, 16]. Third, while our team of psychiatrists made significant efforts to manually review all key words, titles, and study descriptions of trials included in this study to confirm their relevance to mental health, there may have been trials excluded from our analysis due to missing or mislabeled keywords in the registry. We consciously excluded some trials within the neurocognitive disorder category, such as Alzheimer’s disease and traumatic brain injury, because we found they overlapped significantly with the neurology literature. However, to our knowledge, we included all other available search terms for the disorders defined by Section II Diagnostic Criteria and Codes in the *DSM-5*. While there are limitations to a categorical versus a dimensional diagnostic system, as is evidenced by the growing number of trials in the registry that study conditions that do not fit a *DSM-5* diagnostic category, the *DSM-5* currently provides the most universally used schema by which mental health disorders are organized [41]. Finally, our study chose to look exclusively at US trials registered in ClinicalTrials.gov. Heterogeneity in international legislation and incentives for trial registration that differ by country were thought to likely confound the interpretation of our results if they were included in the sample. Consequently, 43.6% of the mental health trials registered in ClinicalTrials.gov from October 1, 2007 to April 30, 2018 were not analyzed in this study, and our results cannot be generalized beyond United States mental health trials. Future analysis will be needed to see how international mental health trials may differ by region and compare to US mental health trials in the registry. There also remains the need to address other important unanswered questions about mental health trials, such as the extent to which publication bias occurs. Others have been able to address this issue using the ClnicalTrials.gov database [42–44].

In summary, this study of the entire portfolio of US mental health trials registered in ClinicalTrials.gov provides clarity to many questions left unanswered by prior analysis of these data [16]. While overall US mental health trials grew at a similar rate to non-psychiatric US trials in the registry, there were significant differences in changes in funders, with more dramatic decreases in industry- and US government- and increase in academic medical center/hospital/other-funders in mental health trials. Features of trial design that provide safeguards against bias, such as blinding and monitoring by a DMC, decreased over time in registered mental health trials, though this may be due to dramatic growth of trials studying behavioral or Other interventions, which often do not lend themselves to blinding or require DMC oversight. There was also a concomitant decline in the registry of mental health trials studying pharmacotherapies. Shifts have occurred in the mental health disorders studied in the registry, with a notable increase in studies of Non-DSM conditions, which may reflect the adoption of the RDoC initiative put forth by NIMH. Despite ethical obligations and policy incentives, trial discontinuation and lack of results reporting remain issues within mental health research. Altogether, we hope our findings foster discussions and collaborations among mental health providers, funding bodies, and other concerned parties to promote the continued development of diverse, well designed, innovative clinical research to improve the care of our patients suffering from mental health disorders.

## Supporting information

S1 Table Medical Subject Heading (MeSH) terms and disease condition terms within ClinicialTrials.gov that were selected to filter trials relevant to mental health. (DOCX)

S2 Table DSM-5 Section II diagnostic criteria and codes and associated diagnoses used to further parse disorders studied in mental health trials. (DOCX)

S3 Table Changes to the initial protocol. (DOCX)

S1 File (DOCX)
