# Supplementary material for: Trends in mental health clinical research: Characterizing the ClinicalTrials.gov registry from 2007–2018
Source: PLoS One. 2020 Jun 5;15(6):e0233996. doi: 10.1371/journal.pone.0233996 (PMC7274444; doi:10.1371/journal.pone.0233996)
Supplement: S1 Table — (DOCX) [file pone.0233996.s001.docx]

**S1 Table. Medical Subject Heading (MeSH) terms and Disease Condition terms within ClinicialTrials.gov that were selected to filter trials relevant to mental health.**

| **MESH** | **Disease Conditions** | | |
| --- | --- | --- | --- |
| adjustment disorders | acute schizophrenia | depressive disorder | pdd |
| affective disorders, psychotic | acute stress disorder | depressive disorder and anxiety disorders | pediatric bipolar disorder |
| affective symptoms | adaptation, psychological | depressive disorder, major | personal satisfaction |
| aggression | addiction | depressive disorder, treatment-resistant | personality |
| agoraphobia | adjustment disorder(s) | depressive disorders | personality disorder(s) |
| akathisia, drug-induced | adolescent - emotional problem | depressive episode | pervasive developmental disorder |
| alcohol abstinence | adolescent behavior | depressive symptoms | phobia, social |
| alcohol amnestic disorder | adolescent depression | diagnosis, psychiatric | phobias |
| alcohol drinking | adolescent substance use | disruptive behavior | phobic disorders |
| alcohol drinking in college | adult attention deficit hyperactivity disorder | disruptive behavior disorder | physical violence |
| alcohol withdrawal delirium | affect | dissociative disorders | physician-patient relations |
| alcohol withdrawal seizures | affective disorders | domestic violence | placebo effect |
| alcoholic intoxication | affective disorders, psychotic | drinking behavior | post (-)traumatic stress disorder(s) (ptsd) |
| alcoholism | affective psychosis, bipolar | driving under the influence | problem behavior |
| amphetamine-related disorders | aggression | drug abuse | problem drinking |
| anhedonia | aggressive behavior | drug addiction | prodromal schizophrenia |
| anorexia | agitation | drug dependence | psychiatric disorder(s) |
| anorexia nervosa | agitation in patients with dementia of the alzheimer's type | drug use | psychiatric hospitalization |
| antisocial personality disorder | agitation, psychomotor | drug use disorders | psychiatry |
| anxiety disorders | agoraphobia | dysthymia | psychological |
| anxiety, separation | alcohol abuse | dysthymic disorder | psychological adjustment |
| asperger syndrome | alcohol addiction | eating behavior | psychological distress |
| attention deficit and disruptive behavior disorders | alcohol consumption | eating behaviors | psychological impact of cancer |
| attention deficit disorder with hyperactivity | alcohol dependence | eating behaviour | psychological stress |
| autism spectrum disorder | alcohol drinking | eating disorder(s) | psychological trauma |
| autistic disorder | alcohol intoxication | ect | psychology, social |
| behavior, addictive | alcohol related disorders | emergence delirium | psychomotor agitation |
| behavioral symptoms | alcohol use | emotion | psychomotor impairment |
| binge drinking | alcohol use disorder(s) | emotion regulation | psychopathology |
| binge-eating disorder | alcohol withdrawal | emotional disorder | psychopathy |
| bipolar and related disorders | alcohol withdrawal syndrome | emotional distress | psychoses |
| bipolar disorder | alcohol; harmful use | emotional disturbances | psychosis |
| body dysmorphic disorders | alcoholic intoxication | emotional intelligence | psychosis nos |
| borderline personality disorder | alcoholism | emotional stress | psychosocial effects of cancer and its treatment |
| bulimia | alcohol-related disorders | emotions | psychosocial problem |
| bulimia nervosa | alexithymia | empathy | psychotic depression |
| bullying | amphetamine addiction | empowerment | psychotic disorder(s) |
| burnout, professional | amphetamine dependence | excessive daytime sleepiness | ptsd |
| cataplexy | amphetamine-related disorders | excessive sleepiness | refractory schizophrenia |
| catatonia | anger | excessive worry | resilience, psychological |
| child behavior disorders | anhedonia | exercise addiction | risk-taking |
| child development disorders, pervasive | anorexia | exploratory behavior | risky sexual behavior |
| child nutrition disorders | anorexia nervosa | family conflict | rumination |
| childhood-onset fluency disorder | antisocial behavior | fatigue syndrome, chronic | schizo affective disorder |
| chorea | anxiety | fear | schizoaffective |
| cocaine-related disorders | anxiety disorder(s) | fear of childbirth | schizoaffective disorder(s) |
| combat disorders | anxiety disorders and symptoms | fear of falling | schizo-affective disorder |
| compassion fatigue | anxiety state | feedback, psychological | schizophrenia |
| compulsive behavior | anxiety symptoms | feeding and eating disorders | schizophrenia and disorders with psychotic features |
| compulsive personality disorder | anxiety, separation | feeding and eating disorders of childhood | schizophrenia and related disorders |
| conduct disorder | apathy | feeding behavior(s) | schizophrenia spectrum and other psychotic disorders |
| conversion disorder | appetitive behavior | feeding disorder | schizophrenic disorders |
| cyclothymic disorder | asperger('s) disorder | feeding intolerance | schizophreniform disorder(s) |
| delirium | asperger('s) syndrome | female sexual arousal disorder | schizotypal personality disorder |
| delusions | attention | female sexual dysfunction | seasonal affective disorder |
| depersonalization | attention deficit | first episode psychosis | selective mutism |
| depression | attention deficit and disruptive behavior disorders | gambling | self esteem |
| depression, postpartum | attention deficit disorder | gambling disorder | self-efficacy |
| depressive disorder | attention deficit disorder(s) with hyperactivity | gambling, pathological | self-esteem |
| depressive disorder, major | attention deficit hyperactivity disorder | gender | self-injurious behavior |
| depressive disorder, treatment-resistant | attention (-/)deficit (-/)hyperactivity disorder(s) (adhd) | generalized anxiety | self-management |
| disruptive, impulse control, and conduct disorders | attitude | generalized anxiety disorder | self-regulation |
| dissociative disorders | attitude to health | grief | separation anxiety disorder |
| drinking behavior | attitudes | habituation | serious mental illness |
| drug-seeking behavior | atypical depression | happiness | severe mental illness |
| dysthymic disorder | auditory hallucinations | hazardous drinking | sex behavior |
| emergence delirium | autism | heavy drinking | sexual abuse |
| encopresis | autism spectrum disorder(s) (asd) | heroin addiction | sexual assault |
| feeding and eating disorders | autistic disorder | heroin dependence | sexual assault and rape |
| feeding and eating disorders of childhood | behavior | hoarding disorder | sexual behavior |
| fibromyalgia | behavior and behavior mechanisms | hypnosis | sexual dysfunctions, psychological |
| firesetting behavior | behavior disorders | hypoactive sexual desire disorder | sexual risk behavior |
| gambling | behavior problem(s) | hypochondriasis | sexual violence |
| gender dysphoria | behavior therapy | impulse control disorders | sexuality |
| hallucinations | behavior, addictive | impulsive behavior | shame |
| heroin dependence | behavior, health | impulsivity | smokeless tobacco use |
| hoarding disorder | behavioral | intermittent explosive disorder | smoking cessation |
| hypochondriasis | behavioral problems | interpersonal relations | social anxiety |
| hysteria | behavioral symptoms | intimate partner violence | social anxiety disorder |
| illness behavior | behaviour | life stress | social behavior |
| illusions | benzodiazepine dependence | loneliness | social cognition |
| impulsive behavior | bereavement | major depression | social interaction |
| inhalant abuse | binge drinking | major depression with psychotic features | social isolation |
| marijuana abuse | binge eating | major depressive disorder | social phobia |
| mental disorders | binge (-)eating disorder | major depressive disorder (mdd) | social psychology |
| mood disorders | bipolar | major depressive disorder, recurrent | social skills |
| morphine dependence | bipolar 1 disorder | major depressive disorders | social stress |
| multiple personality disorder | bipolar affective disorder | major depressive episode | somatization disorder(s) |
| neuroleptic malignant syndrome | bipolar depression | mania | spasmodic torticollis |
| night terrors | bipolar disorder(s) | manic disorder | specific phobia |
| obsessive behavior | bipolar i depression | marijuana abuse | stimulant abuse |
| obsessive-compulsive disorder | bipolar i disorder | marijuana dependence | stimulant dependence |
| opioid-related disorders | bipolar ii disorder | marijuana smoking | stress |
| passive-aggressive personality disorder | body dysmorphic disorder(s) | marijuana use | stress disorder(s) |
| personality disorders | body image | maternal behavior | stress disorders, post (-)traumatic |
| phobia, social | borderline personality disorder | maternal depression | stress disorders, traumatic |
| phobic disorders | bulimia | maternal-fetal relations | stress related disorder |
| pica | bulimia nervosa | mdd | stress response |
| premenstrual dysphoric disorder | bullying | meditation | stress, emotional |
| problem behavior | burnout | mental disorder(s) | stress, psychological |
| psychological trauma | burnout syndrome | mental disorders diagnosed in childhood | substance abuse |
| psychomotor agitation | burnout, professional | mental fatigue | substance abuse disorder |
| psychomotor disorders | cannabis abuse | mental health | substance abuse problem |
| psychophysiologic disorders | cannabis dependence | mental health disorder(s) | substance abuse, intravenous |
| psychoses, substance-induced | cannabis use | mental health impairment | substance addiction |
| psychotic disorders | cannabis use disorder | mental health wellness | substance dependence |
| restless legs syndrome | child abuse | mental illness | substance related disorders |
| seasonal affective disorder | child behavior | mental stress | substance use |
| self mutilation | child behavior disorders | mental well-being | substance use disorder(s) |
| self-injurious behavior | child behavior problems | mindfulness | substance withdrawal syndrome |
| serotonin syndrome | child development | mood | substance-related disorders |
| sexual dysfunctions, psychological | child development disorders, pervasive | mood disorder(s) | suicidal and self-injurious behavior |
| shared paranoid disorder | child maltreatment | mother-child relations | suicidal behavior |
| sleep deprivation | child neglect | motivation | suicidal ideation |
| somatoform disorders | chronic tic disorder | motivational interviewing | suicidality |
| specific learning disorder | clinical high risk for psychosis | music therapy | suicide |
| stereotypic movement disorder | cocaine abuse | narcotic abuse | suicide and self-harm |
| stress disorders, post-traumatic | cocaine addiction | narcotic use | suicide attempt |
| stress disorders, traumatic | cocaine dependence | neurobehavioral manifestations | suicide prevention |
| stress disorders, traumatic, acute | cocaine related disorders | neuropsychology | suicide, attempted |
| stress, psychological | cocaine use disorder(s) | nicotine addiction | tardive dyskinesia |
| substance abuse, intravenous | cocaine-related disorders | nicotine dependence | tic disorder(s) |
| substance withdrawal syndrome | college student drinking | nicotine dependence, cigarettes | tobacco addiction |
| substance-related disorders | combat disorders | nicotine use disorder | tobacco dependence |
| suicidal ideation | compassion | nicotine withdrawal | tobacco smoking |
| suicide | compulsive behavior | obsessive (-)compulsive disorder (ocd) | tobacco smoking behavior |
| suicide, attempted | conduct disorder | ocd | tobacco use |
| tardive dyskinesia | conversion disorder | opiate addiction | tobacco use cessation |
| tic disorders | coping | opiate dependence | tobacco use disorder |
| tobacco use disorder | coping behavior | opiate withdrawal syndrome | torticollis |
| tourette syndrome | coping skills | opioid abuse | tourette disorder |
| trauma and stressor related disorders | counseling | opioid addiction | tourette syndrome |
| underage drinking | criminogenic thinking | opioid dependence | tourette's disorder |
|  | cumulative trauma disorders | opioid dependency | tourette's syndrome |
|  | delinquency | opioid use disorder(s) | transsexualism |
|  | delirium | opioid withdrawal | trauma, psychological |
|  | delirium in old age | opioid-related disorders | treatment resistant depression |
|  | delirium on emergence | opioid-use disorder | treatment resistant major depressive disorder |
|  | delirium, dementia, amnestic, cognitive disorders | oppositional defiant disorder | treatment-resistant depression |
|  | delusional disorder | pain associated with fibromyalgia | treatment-resistant schizophrenia |
|  | dependence | pain perception | unipolar depression |
|  | depression | panic attacks | violence |
|  | depression, anxiety | panic disorder | violence, non-accidental |
|  | depression, bipolar | parent-child relations | well (-)being |
|  | depression, postpartum | parenting |  |
|  | depression, unipolar | pathological gambling |  |
