# Supplementary material for: Trends in mental health clinical research: Characterizing the ClinicalTrials.gov registry from 2007–2018
Source: PLoS One. 2020 Jun 5;15(6):e0233996. doi: 10.1371/journal.pone.0233996 (PMC7274444; doi:10.1371/journal.pone.0233996)
Supplement: S2 Table — (DOCX) [file pone.0233996.s002.docx]

**S2 Table. DSM-5 Section II Diagnostic Criteria and Codes and associated diagnoses used to further parse disorders studied in mental health trials.**

| **Section II Diagnostic Criteria and Codes** | **Disorders Included** |
| --- | --- |
| Neurodevelopmental Disorders | Global Developmental Delay, Unspecified Intellectual Disability, Communication Disorders, Autism Spectrum Disorder, Attention Deficit/Hyperactivity Disorder, Specific Learning Disorder, Motor Disorders, Tic Disorders |
| Schizophrenia Spectrum and Other Psychotic Disorders | Delusional Disorder, Brief Psychotic Disorder, Schizophreniform Disorder, Schizophrenia, Schizoaffective Disorder, Substance/Medication-Induced Psychotic Disorder, Psychosis NOS, Catatonia |
| Bipolar and Related Disorders | Manic Episode, Hypomanic Episode, Major Depressive Episode, Bipolar I Disorder, Bipolar II Disorder, Cyclothymic Disorder, Substance-Induced Bipolar Disorder |
| Depressive Disorders | Major Depressive Disorder, Persistent Depressive Disorder, Premenstral Dysphoric Disorder, Substance/Medication-Induced Depressive Disorder, Depressive Disorder Due to Another Medical Condition, Unspecified Depressive Disorder |
| Anxiety Disorders | Selective Mutism, Specific Phobia, Social Anxiety Disorder, Panic Disorder, Agoraphobia, Generalized Anxiety Disorder, Substance/Medication-Induced Anxiety Disorder, Anxiety Disorder Due to Another Medical Condition, Unspecified Anxiety Disorder |
| Obsessive-Compulsive and Related Disorders | Body Dysmorphic Disorder, Hoarding Disorder, Trichotillomania, Excoriation, Substance/Medication-Induced Obsessive-Compulsive and Related Disorder, Obsessive-Compulsive and Related Disorder Due to Another Medical Condition, Other Specified Obsessive-Compulsive and Related Disorder, Unspecified Obsessive-Compulsive and Related Disorder |
| Trauma- and Stressor-Related Disorders | Disinhibited Social Engagement Disorder, Posttraumatic Stress Disorder, Acute Stress Disorder, Adjustment Disorders, Other Specified Trauma- and Stressor-Related Disorders, Unspecified Trauma- and Stressor-Related Disorders |
| Dissociative Disorders | Dissociative Amnesia, Depersonalization/Derealization Disorder, Other Specified Dissociative Disorders, Unspecified Dissociative Disorder |
| Somatic Symptom and Related Disorders | Illness Anxiety Disorder, Conversion Disorder, Factitious Disorder, Factitious Disorder Imposed on Another, Other Specified Somatic Symptom and Related Disorder, Unspecified Somatic Symptom and Related Disorder |
| Feeding and Eating Disorders | Rumination Disorder, Avoidant/Restrictive Food Intake Disorder, Anorexia Nervosa, Bulimia Nervosa, Binge-Eating Disorder, Other Specified Feeding or Eating Disorder, Unspecified Feeding or Eating Disorder |
| Sleep Disorders | Breathing-Related Sleep Disorder and Sleep-Wake Disorders clustered together: Hypersomnolence Disorder, Narcolepsy, Central Sleep Apnea, Sleep-Related Hypoventilation, Circadian Rhythm Sleep-Wake Disorders |
| Parasomnias | Nightmare Disorder, Rapid Eye Movement Sleep Behavior Disorder, Restless Legs Syndrome, Substance/Medication-Induced Sleep Disorder, Other Specified Insomnia Disorder, Unspecified Insomnia Disorder, Other Specified Hypersomnolence Disorder, Unspecified Hypersomnolence Disorder, Other Specified Sleep-Wake Disorder, Unspecified Sleep-Wake Disorder |
| Sexual Dysfunctions | Erectile Disorder, Female Orgasmic Disorder, Female Sexual Interest/Arousal Disorder, Genito-Pelvic Pain/Penetration Disorder, Male Hypoactive Sexual Desire Disorder, Premature (Early) Ejaculation, Substance/Medication-Induced Sexual Dysfunction, Other Specified Sexual Dysfunction, Unspecified Sexual Dysfunction |
| Gender Dysphoria | Other Specified Gender Dysphoria, Unspecified Gender Dysphoria |
| Disruptive, Impulse-Control, and Conduct Disorders | Intermittent Explosive Disorder, Conduct Disorder, Antisocial Personality Disorder, Pyromania, Kleptomania, Other Specified Disruptive, Impulse-Control, and Conduct Disorder, Unspecified Disruptive, Impulse Control, and Conduct Disorder |
| Substance-Related and Addictive Disorders | Substance-Related Disorders, Alcohol-Related Disorders, Caffeine-Related Disorders, Cannabis-Related Disorders, Hallucinogen-Related Disorders, Inhalant-Related Disorders, Opioid-Related Disorders, Sedative, Hypnotic-, or Anxiolytic-Related Disorders, Stimulant-Related Disorders, Tobacco-Related Disorders, Other (or Unknown) Substance-Related Disorders |
| Neurocognitive Disorders | Delirium, Other Specified Delirium, Unspecified Delirium. Of note, Major and Minor Neurocognitive Disorders, such as Alzheimer’s Disease and Traumatic Brain Injury were not included in this analysis, as these conditions were found to have too much overlap with the neurology literature. |
| Personality Disorders | Cluster A Personality Disorders, Cluster B Personality Disorders B, Cluster C Personality Disorders, and Other Personality Disorders |
| Paraphilic Disorders | Exhibitionistic Disorder, Frotteuristic Disorder, Sexual Masochism Disorder, Sexual Sadism Disorder, Pedophilic Disorder, Fetishistic Disorder, Transvestic Disorder, Other Specified Paraphilic Disorder, Unspecified Paraphilic Disorder |
| Non-DSM | Conditions that did not fall into any of the above categories (e.g. suicide, wellness, burnout). |
