# Supplementary material for: Trends in mental health clinical research: Characterizing the ClinicalTrials.gov registry from 2007–2018
Source: PLoS One. 2020 Jun 5;15(6):e0233996. doi: 10.1371/journal.pone.0233996 (PMC7274444; doi:10.1371/journal.pone.0233996)
Supplement: S3 Table — (DOCX) [file pone.0233996.s003.docx]

**S3 Table. Changes to the initial protocol.**

| **Changes to the original protocol** | **Rationale for changes** |
| --- | --- |
| (1) We limited our analysis to only United States trials in our revised protocol. | Initially we included United States and international trials together in our sample to capture the full range of interventional mental health studies in the ClinicalTrials.gov registry. This practice has been used in several previous analyses of the ClinicalTrials.gov registry [7, 10, 14]. However, our reviewers raised valid concerns that including international trials likely introduced significant bias into our sample, as trials from different countries are subject to different laws and incentives for reporting. We considered including a stratified analysis of US and international mental health trials in our revisions; however, this raised two primary concerns. First, because trial registration and results reporting vary significantly by region, it would be difficult to know whether comparisons between US and non-US mental health trials were due to differences in the research conducted in these regions or regional differences in the types of trials registered in ClinicalTrials.gov. Moreover, the international category itself is composed of trials from over 200 countries, and to cluster these trials together in an analysis seemed similarly prone to bias as our initial analysis that clustered US trials with international studies. Second, because most non-US trials did not receive US government funding (i.e. only 1.8% of international mental health trials in our sample received US government funding), and because international governmental funders are listed in ClinicalTrials.gov as ‘Other,’ comparing US and non-US trial funding would be difficult to interpret. Therefore, we decided to keep our study limited to US trials, which we think leads to more interpretable results with fewer sources of confounding. We cite the need for analysis of international mental health trials as an area for further study in our Discussion section. Such efforts should ideally pool data from multiple registries to better represent the diverse national registries that are now available. The reduction of our sample to include only US trials impacted our power calculations, which is reflected in the revised protocol; however, our overall test power was minimally affected (i.e. remained ~1.00) despite this change. |
| (2) We combined the ClinicalTrials.gov categories 'NIH' and 'US Fed' to create a new funder category 'US Govt.' | Initially we had excluded 'US Fed'-funded studies from our analysis, as they comprise only 3.5% of studies in the ClinicalTrials.gov registry and were excluded for this reason in other analyses of the ClinicalTrials.gov registry [14]. However, our reviewers encouraged us to include both 'NIH' and 'US Fed' studies to form a new funder category called 'US Govt,' as has been done in some other analyses of the database to better capture changes in US government-funded trials [16]. Our protocol was adjusted to account for this change. |
| (3) Other than looking at disorders and interventions studied by funder type, we removed analysis of trial characteristics stratified by funder type from our revised analysis. | In our initial manuscript we included a table stratifying trial characteristics by funder type for mental health trials in the ClinicalTrials.gov registry from 2007-2018. However, since performing this analysis, the study by Arnow et al., 2019 was published, which conducted a very similar analysis for mental health trials in the ClinicalTrials.gov registry from 2007 – 2014 [16]. Therefore, so as not to duplicate their work, we replaced this table with a table of trial characteristics stratified by mental health trials and non-mental health trials in the ClinicalTrials.gov registry. This analysis had been part of our original protocol, but the results were previously only reported in the text of the Results section. |
| (4) We removed analysis of enrollment from our revised protocol. | We found that that the majority of completed (95.8%) and prematurely stopped (90.1%) trials reported actual enrollment to ClinicalTrials.gov, with the remainder reporting anticipated/estimated enrollment. For the majority of non-completed trials (comprising 34% of the sample), actual enrollment was not reported. We initially included trials that reported either actual or anticipated/estimated enrollment (none reported both); however, our reviewers advised that estimated enrollment is an unreliable metric, as many trials do not meet this projected enrollment number. Because studying only the trials that reported actual enrollment would introduce significant bias into our analysis, we removed almost all discussion of enrollment from our revised analysis. The only instance where we preserved discussion of enrollment was regarding the number of participants enrolled in discontinued trials. Here enrollment refers to actual enrollment. |
| (5) We grouped Phase 1/2 trials with Phase 2 trials into a new combined category, and we grouped Phase 2/3 trials with Phase 3 trials into a new combined category. | Given that Phase 1/2 and Phase 2/3 trials did not clearly fit an FDA-defined phase, our initial protocol grouped these trials into the category ‘Not Applicable’ (NA) (comprising 9.6% of this category). However, our reviewers suggested that, given Phase 1/2 trials reach Phase 2 status and Phase 2/3 trials reach Phase 3 status, we group these trials together, respectively, rather than with NA. Therefore, in our revised analysis, our new phase categories are Phase 1, Phase 1/2 - 2, Phase 2/3 - 3, Phase 4, and NA. |
| (6) We extended the period of time for results reporting to the ClinicalTrials.gov registry from 2 to 3 years post-trial completion. | The FDAAA Section 801 and Final Rule require reporting of results for applicable trials within 12 months, also allowing for up to a 2-year extension for certain trials (i.e. within 3 years of trial completion). We initially chose to examine reporting within two years of completion to account for the 12-month reporting period and the mid-point of the available extension time a trial could be extended. We selected this time period not to investigate legal compliance, but rather to assess timely reporting of results even for trials that were not legally obligated. For this reason we limited our initial analysis to trials that were completed by April 30, 2016 to provide a full two years from our download date (April 30, 2018). However, our reviewers noted that because we cannot determine which trials used the full 2-year extension (i.e. 3 years from trial completion), incorporating a 3-year reporting cutoff after a trial’s primary completion date would be a more robust analysis. We agreed with the benefits of a 3-year cutoff, and for this reason, we limited our revised analysis of results reporting to trials completed by April 30, 2015. Our reviewers also advised us to assess results reporting even beyond this 3-year period. This analysis was conducted, but we did not identify significant differences between this model and the model with the strict 3-year cutoff. |
| (7) We added an analysis of intervention type to our protocol. | Our reviewers asked that we include an analysis of trial intervention types, which is a variable provided in the ClinicalTrials.gov database. We added this to our revised analysis. |
| (8) We included a new reference (Arnow et al., 2019)[16]. | The manuscript by Arnow and colleagues was published after we wrote our protocol and completed our initial analysis. We have added it to our revised protocol because this paper was fundamental to why we subsequently adjusted several aspects of our analysis in an effort to remove the parts of our investigation that were already explored within their findings. |
