# Supplementary material for: Trends in mental health clinical research: Characterizing the ClinicalTrials.gov registry from 2007–2018
Source: PLoS One. 2020 Jun 5;15(6):e0233996. doi: 10.1371/journal.pone.0233996 (PMC7274444; doi:10.1371/journal.pone.0233996)
Supplement: S1 File — (DOCX) [file pone.0233996.s004.docx]

**Study Protocol**

1. **Background:**

The ClinicalTrials.gov database was first released in 2000 to facilitate the monitoring and study of clinical trials. Reporting of results into the database was first required after the passing of the FDA Amendments Act of 2007 (FDAAA). The passing of the Final Rule clarified which trials were obligated to register and submit results, and went into effect in January 2017.

Several studies have examined this database to answer valuable questions about the clinical research enterprise. Our analysis will explore the state of mental health clinical research within the ClinicalTrials.gov database. Several others have utilized this database to assess the state of research, for example looking at Oncological trials [1]. Mental health related trials have been less commonly studied using this database. The earliest large-scale effort included a review of trials between 2007-2010 by Califf and colleagues, which examined Mental Health trials along with trials in Cardiology and Oncology [2]. Most recently, Arnow and colleagues published an analysis of mental health clinical trials registered in ClinicalTrials.gov from 2007-2014 to identify differences in trial characteristics, such as trial design, disorders studied, and interventions tested, as stratified by funder type [3]. We wish to perform a more expansive analysis including more contemporary trials in Mental Health to explore trends over time and comparisons to non-mental health trials. We wish to also characterize subgroups within the field, and understand how clinical trial characteristics like funding source and trial design influence crucial events like reporting of results and early discontinuation of a trial.

1. **Study Objectives:**
2. Primary Objective:
   1. To assess the funding, trial design, disease focus, and intervention type of mental health trials in the ClinicalTrials.gov database between 2007 and 2018
3. Secondary Objectives:
   1. To Identify major differences in clinical trial design features between trials that involve mental health and all other trials in the database.
   2. To determine any trial characteristics (e.g. funding or design) that influence outcomes of interest, particularly whether results are reported or whether the trial discontinued early.
   3. Research Questions:

Research Question #1: How many new United States trials in mental health are submitted each year and is this quantity consistently increasing or decreasing?

Research Question #2: Is there a difference in the general design of US clinical trials over time (e.g. use of blinding, randomization, multiple facilities)?

Research Question #3: What are the disorders and interventions that are the focus of US mental health related trials, and how is this different depending on who is funding the trial?

Research Question #4: Do any of the general trial characteristics predict the outcome of whether a US trial will report their results to the ClinicalTrials.gov database?

Research Question #5: Do any of the general trial characteristics predict the outcome of whether a US trial will discontinue early?

- 1. Specific Aims:

Specific Aim #1: To identify US mental health trials within the ClinicalTrials.gov database and determine their growth pattern and changes in trial characteristics (e.g. use of blinding, randomization, multiple facilities).

Specific Aim #2: To use trial records to identify which mental health disorders and interventions are studied within the registry, and to identify any differences between Industry, NIH / U.S. Fed, and Other funders of US trials.

Specific Aim #3: To examine the association between US trial characteristics, including trial funder, design, and disease focus, on whether a trial’s results were posted to the ClinicalTrials.gov results database.

Specific Aim #4: To examine the association between US trial characteristics, including trial funder, design, and disease focus, on whether a trial discontinued early.

- 1. Hypotheses:

Hypothesis #1: The number of US mental health trials has not grown over time.

Hypothesis #2: US mental health trials employ less rigorous design features (use of blinding, randomization, multiple arms, multiple sites) than US trials in the rest of the database.

Hypothesis #3: US mental health trials will disproportionately focus on depressive disorders, anxiety disorders, and substance use disorders, and there will be no difference between the different funding categories.

Hypothesis #4: US industry-funded trials will be least likely to report results, and trials with more rigorous parameters (e.g. blinding, randomization, multi-arm) will be most likely to report results.

Hypothesis #5: US industry-funded trials will be the most likely to discontinue early, and use of data monitoring committees will also be associated with earlier discontinuation.

1. **Methods**

3.1 Study Type and Design:

We will use a retrospective, cross-sectional analysis of all US clinical trials in the ClinicalTrials.gov database. Given that we wish to assess the present state of clinical trials in mental health trials, we believe our cross-sectional approach is appropriate and will include both historically completed trials and those which are still active.

3.2 Data Source:

We will access records from ClinicalTrials.gov using the Aggregate Analysis of ClinicalTrials.gov (AACT), a relational database of publicly available ClinicalTrials.gov data.

3.3 Study Population:

We focus our analysis on US mental health trials. These we will identify by manually reviewing all medical subject headings (MeSH) and Disease Condition Terms contained in the entire ClinicalTrials.gov database. These are applied using an algorithm by the National Library of Medicine (NLM), which analyzes text in the trial record to determine the relevant MeSH terms associated with the trial. We will identify all MeSH terms that are related to mental health using review by a team of psychiatrists. To reduce the risk of misclassification bias, we will manually review all trial records using their official title and study description to confirm if they are relevant to mental health.

We include trials submitted from October 1, 2007. We chose this date due to the passing of the FDAAA on September 27, 2007. We downloaded records on April 30, 2018. We will exclude any trial which is not interventional (i.e. observational studies, which can also be registered in the ClinicalTrials.gov database).

3.4 Variable Definition:

We will analyze the following 13 variables, as clarified in the table below: date of submission, primary objective of the intervention, trial phase, number of arms, use of blinding, use of randomization, oversight by a data monitoring committee (DMC), number of sites, funder (i.e. lead sponsor and collaborator class to determine all funders involved), trial status, disorder categories studied, intervention studied, and results reporting status. With the exception of mental disorder focus, all variables will be downloaded directly from the AACT.

| **Domain** | **Variable** | **Original Values** |
| --- | --- | --- |
| Outcome | Results reporting | 0: No 1: Yes |
| Outcome | Study Status | 0: Ongoing 1: Stopped Early 2: Completed 9: Unknown |
| Covariate | Study first submitted date | 2007-2018 |
| Covariate | Primary purpose | 0: Treatment 1: Basic Science 2: Prevention 3: Device feasibility 4: Diagnostic 5: Health Services Research 6: Screening 7: Supportive Care 8: Other |
| Covariate | Phase | 0: Early Phase 1 1: Phase 1 2: Phase 1/Phase 2 3: Phase 2 4: Phase 2/Phase 3 5: Phase 3 6: Phase 4 7: N/A |
| Covariate | Number of Arms | 1-28 |
| Covariate | Masking | 0: None 1: Single 2: Double |
| Covariate | Use of randomization | 0: No randomization 1: Randomization |
| Covariate | Oversight by a DMC | 0: No 1: Yes |
| Covariate | Number of Sites | 1-3511 |
| Covariate | Lead agency class (Sponsor) | 0: Industry 1: NIH 2: U.S. Fed 3: Other |
| Covariate | Collaborator Class | 0: Industry 1: NIH 2: U.S. Fed 3: Other |
| Covariate | Intervention Type | 0: Behavioral  1: Drug  2: Device  3: Procedure  4: Dietary supplement  5: Radiation  6: Biological  7: Genetic  8: Other |
| ~~Covariate~~ | ~~Enrollment~~ | ~~0-1,951,060~~ |
| Covariate | DSM Category | See Table Below |

For analysis of phase, we will combine Early Phase 1 and Phase 1 into ‘Phase 1.’ Phase 1/2 – 2’ was generated by grouping the ClinicalTrials.gov categories Phase 1/2 and Phase 2. ‘Phase 2/3 – 3’ was generated by grouping the ClinicalTrials.gov categories Phase 2/3 and Phase 3. ‘Phase 4’ and ‘Not Applicable’ were taken directly from these corresponding categories in ClinicalTrials.gov.

For analysis of Funder, we combined U.S. Fed with NIH into a new category ‘US Govt,’ as has been done in prior analyses of the ClinicalTrials.gov database [3].

To define our Diagnostic and Statistical Manual (DSM) categories, we will use *DSM-5* Section II Diagnostic Criteria and Codes designations below, and any disorders not defined within *DSM-5* will be assigned the label “Non-DSM” [4].

| **Section II Diagnostic Criteria and Codes** | **Disorders Included** |
| --- | --- |
| Neurodevelopmental Disorders | Global Developmental Delay, Unspecified Intellectual Disability, Communication Disorders, Autism Spectrum Disorder, Attention Deficit/Hyperactivity Disorder, Specific Learning Disorder, Motor Disorders, Tic Disorders |
| Schizophrenia Spectrum and Other Psychotic Disorders | Delusional Disorder, Brief Psychotic Disorder, Schizophreniform Disorder, Schizophrenia, Schizoaffective Disorder, Substance/Medication-Induced Psychotic Disorder, Psychosis NOS, Catatonia |
| Bipolar and Related Disorders | Manic Episode, Hypomanic Episode, Major Depressive Episode, Bipolar I Disorder, Bipolar II Disorder, Cyclothymic Disorder, Substance-Induced Bipolar Disorder |
| Depressive Disorders | Major Depressive Disorder, Persistent Depressive Disorder, Premenstral Dysphoric Disorder, Substance/Medication-Induced Depressive Disorder, Depressive Disorder Due to Another Medical Condition, Unspecified Depressive Disorder |
| Anxiety Disorders | Selective Mutism, Specific Phobia, Social Anxiety Disorder, Panic Disorder, Agoraphobia, Generalized Anxiety Disorder, Substance/Medication-Induced Anxiety Disorder, Anxiety Disorder Due to Another Medical Condition, Unspecified Anxiety Disorder |
| Obsessive-Compulsive and Related Disorders | Body Dysmorphic Disorder, Hoarding Disorder, Trichotillomania, Excoriation, Substance/Medication-Induced Obsessive-Compulsive and Related Disorder, Obsessive-Compulsive and Related Disorder Due to Another Medical Condition, Other Specified Obsessive-Compulsive and Related Disorder, Unspecified Obsessive-Compulsive and Related Disorder |
| Trauma- and Stressor-Related Disorders | Disinhibited Social Engagement Disorder, Posttraumatic Stress Disorder, Acute Stress Disorder, Adjustment Disorders, Other Specified Trauma- and Stressor-Related Disorders, Unspecified Trauma- and Stressor-Related Disorders |
| Dissociative Disorders | Dissociative Amnesia, Depersonalization/Derealization Disorder, Other Specified Dissociative Disorders, Unspecified Dissociative Disorder |
| Somatic Symptom and Related Disorders | Illness Anxiety Disorder, Conversion Disorder, Factitious Disorder, Factitious Disorder Imposed on Another, Other Specified Somatic Symptom and Related Disorder, Unspecified Somatic Symptom and Related Disorder |
| Feeding and Eating Disorders | Rumination Disorder, Avoidant/Restrictive Food Intake Disorder, Anorexia Nervosa, Bulimia Nervosa, Binge-Eating Disorder, Other Specified Feeding or Eating Disorder, Unspecified Feeding or Eating Disorder |
| Sleep Disorders | Breathing-Related Sleep Disorder and Sleep-Wake Disorders clustered together: Hypersomnolence Disorder, Narcolepsy, Central Sleep Apnea, Sleep-Related Hypoventilation, Circadian Rhythm Sleep-Wake Disorders |
| Parasomnias | Nightmare Disorder, Rapid Eye Movement Sleep Behavior Disorder, Restless Legs Syndrome, Substance/Medication-Induced Sleep Disorder, Other Specified Insomnia Disorder, Unspecified Insomnia Disorder, Other Specified Hypersomnolence Disorder, Unspecified Hypersomnolence Disorder, Other Specified Sleep-Wake Disorder, Unspecified Sleep-Wake Disorder |
| Sexual Dysfunctions | Erectile Disorder, Female Orgasmic Disorder, Female Sexual Interest/Arousal Disorder, Genito-Pelvic Pain/Penetration Disorder, Male Hypoactive Sexual Desire Disorder, Premature (Early) Ejaculation, Substance/Medication-Induced Sexual Dysfunction, Other Specified Sexual Dysfunction, Unspecified Sexual Dysfunction |
| Gender Dysphoria | Other Specified Gender Dysphoria, Unspecified Gender Dysphoria |
| Disruptive, Impulse-Control, and Conduct Disorders | Intermittent Explosive Disorder, Conduct Disorder, Antisocial Personality Disorder, Pyromania, Kleptomania, Other Specified Disruptive, Impulse-Control, and Conduct Disorder, Unspecified Disruptive, Impulse Control, and Conduct Disorder |
| Substance-Related and Addictive Disorders | Substance-Related Disorders, Alcohol-Related Disorders, Caffeine-Related Disorders, Cannabis-Related Disorders, Hallucinogen-Related Disorders, Inhalant-Related Disorders, Opioid-Related Disorders, Sedative, Hypnotic-, or Anxiolytic-Related Disorders, Stimulant-Related Disorders, Tobacco-Related Disorders, Other (or Unknown) Substance-Related Disorders |
| Neurocognitive Disorders | Delirium, Other Specified Delirium, Unspecified Delirium. Of note, Major and Minor Neurocognitive Disorders, such as Alzheimer’s Disease and Traumatic Brain Injury were not included in this analysis, as these conditions were found to have too much overlap with the neurology literature. |
| Personality Disorders | Cluster A Personality Disorders, Cluster B Personality Disorders B, Cluster C Personality Disorders, and Other Personality Disorders |
| Paraphilic Disorders | Exhibitionistic Disorder, Frotteuristic Disorder, Sexual Masochism Disorder, Sexual Sadism Disorder, Pedophilic Disorder, Fetishistic Disorder, Transvestic Disorder, Other Specified Paraphilic Disorder, Unspecified Paraphilic Disorder |
| Non-DSM | Conditions that did not fall into any of the above categories (e.g. suicide, wellness, burnout). |

3.5 Sample Size:

A previous analysis of clinical trials from 2007-2010 identified 2,338 North American interventional mental health trials. At this time the database contained 96,346 studies. At the time that we downloaded our data, there were 274,029 studies as of April 30, 2018. This represents an additional 177,683 clinical studies. We estimate that the relative proportion of North American mental health trials has not changed, which would suggest that there are an additional 4,312 interventional mental health trials between 2010 and when we downloaded our records (2018), giving a total sample size of approximately 6,650 North American mental health trials. We assume that the majority of these trials will be from the United States of America, though the exact proportion of US trials was not reported in this earlier analysis. Assuming 90% of these North American trials were US trials, our final approximated sample size of US mental health trials is 5,985 trials. Due to the large number within our sample, for our main outcome, using chi-square analysis, assuming 5,985 trials, 19 degrees of freedom (the maximum for any of our covariates), an α of 0.001, and a small effect size (w = 0.1), our test power is ~1.00.

3.6 Statistical Analysis Plan:

[3.6.1 – Data Management]:

All data will be accessed by download from the AACT SQL database and stored on a research laptop. All data cleaning will be conducted using R version 3.5.0. We will export trial records into excel tables for manual review for mental health disorder assignment. Otherwise all labeling will be done in R.

[3.6.2 – Descriptive Statistics]:

All tests will be two-sided with α = 0.001. We will use Chi-square tests for all categorical data. We will quantify annual change over time using the average annual growth rate (AAGR) and the compound annual growth rate (CAGR). The formula for AAGR is:

$$AAGR=\frac{GR_{1}+GR_{2}+\ldots+GR_{n}}{N}$$

Where N = number of years, GR_A_ = growth rate in year 1, GR_b_ = growth rate in year 2, and GR_n_ = growth rate in the final year.

The formula for CAGR is:

$${CAGR=\left( \frac{Quantity in final year}{\left( Quantity in first year \right)} \right)}^{\frac{1}{\# of years}}-1$$

We will assess for statistical significance of monotonic trends over time using the Mann-Kendall test. We will also create a binary time variable to assess for changes between an early and late period (defined using the mid-point between our sample period).

[3.6.2 – Inferential Statistics]:

We utilize logistic regression to assess association of covariates with the outcome of results reporting. All covariates are assessed to produce unadjusted odds-ratios. We additionally include all covariates regardless of their individual significance in a fully-adjusted model to assess association with results reporting. We include all covariates because our aim is to conduct an exploratory analysis that fully controls for all covariates of interest. We will only include completed trials in this analysis. We will also observe results reporting at 3 years of completion to facilitate correlation with trials that may have a legal requirement to report results due to the FDAAA and final rule (which requires reporting after 1 year, with the option to extend an additional 1-2 years). We therefore will exclude from this analysis any trial that did not complete before April 30, 2015 to allow a full 3 years to determine if their results were reported.

We utilize cox regression to assess association of covariates with the outcome of early termination. For this analysis we consider events to be any trial with status of “Stopped early”, and we censor trials that either completed without early termination or that were ongoing at the date of our download. Trials with unknown status will be excluded from the analysis. For all inferential statistics we exclude trials that do not have complete records for our covariates of interest to avoid missing data.

1. **Study Limitations**

4.1 Data Source:

While the ClinicalTrials.gov repository is one of the largest in the world, it is not complete, and many trials are registered in other registries or simply not registered. Many USA trials are not required to register or report results. In addition to the subset of trials not present in the database, some trials, which are in the database do not have complete records and are missing specific elements (e.g. do not report funder). This may limit the accuracy of our findings and also may limit the generalizability of our findings beyond the ClinicalTrials.gov database.

4.2 Study Design:

Because DSM disorders will be assigned by manual review by a team of psychiatrists, it is possible that some trials could be labeled differently if observed by a different psychiatrist. Our team will collectively reviewed a set of 250 trials to allow synchronization of definitions and confusing cases, and unclear scenarios will be reviewed by the lead authors. However, it is still possible that some labeling decisions may shift the representation of different disorders in the record, which could be more impactful for disorders which are infrequent for the database.

4.3 Statistical Analyses:

Due to our anticipated large cohort, we suspect that some results may reach our threshold for significance, α = 0.001 due to chance. Because this is an exploratory analysis meant to identify important trends within our field, we do not adjust for multiple hypothesis testing, which could also further limit the true statistical significance of our results.

1. **Discussion**

This study will potentially identify major trends in the production of United States mental health clinical research. Because much of mental health funding, research, and practice are targeted to specific disorders, we hope to provide a nuanced look at the distribution of various DSM disorders among our field’s research portfolio. Further dividing that data by funding source will provide a closer look at which organizations are driving the clinical research for specific disorders, and the over- or under-representation of various disorders should be discussed with key stakeholders to determine if any changes are warranted.

Dissemination of trial results is crucial for advancing science and respecting the large investments that are required (by patients, clinicians, and other institutions) for clinical trials to continue as an important part of the research ecosystem. Relatedly, these same resources are poorly allocated when trials discontinue early. By identifying features associated with results reporting and trial discontinuation, we hope to provide potential mechanisms that may become targets for policy or other efforts to improve these outcomes and enhance the quality of mental health research available for the future.

1. **References**

1. Liu X, Zhang Y, Tang L, et al. Characteristics of radiotherapy trials compared with other oncological clinical trials in the past 10 years. JAMA Oncology. 2018. doi: 10.1001/jamaoncol.2018.0887.

2. Califf RM, Zarin DA, Kramer JM, Sherman RE, Aberle LH, Tasneem A. Characteristics of clinical trials registered in clinicaltrials.gov, 2007-2010. JAMA. 2012;307(17):1838-47. doi: 10.1001/jama.2012.3424.

3. Arnow KD, King AC, Wagner TH. Characteristics of mental health trials registered in ClinicalTrials.gov. Psychiatry Res. 2019;281:112552. Epub 2019/10/19. doi: 10.1016/j.psychres.2019.112552. PubMed PMID: 31627072.

4. Copyright. Diagnostic and Statistical Manual of Mental Disorders. DSM Library: American Psychiatric Association; 2013.
